# DNA Nanoarray for Multiplexed RNA Detection With Single‐Molecule Readout

**DOI:** 10.1002/advs.75319

**Published:** 2026-04-28

**Authors:** Yunxuan Li, Yesheng Wang, Chunmiao Yu, Siyu Hou, Elli Mylona, Plamena Naydenova, Jingyuan Zhao, Hong Yuan, Jingsong Duan, Hendrik F. P. Runge, Stephen Baker, Jinbo Zhu, Ulrich F. Keyser

**Affiliations:** ^1^ Cavendish Laboratory University of Cambridge Cambridge UK; ^2^ School of Biomedical Engineering Faculty of Medicine Dalian University of Technology Dalian China; ^3^ Cambridge Institute of Therapeutic Immunology & Infectious Disease (CITIID) Jeffery Cheah Biomedical Centre Cambridge Biomedical Campus University of Cambridge Cambridge UK; ^4^ Central Hospital of Dalian University of Technology (Dalian Municipal Central Hospital) Dalian China; ^5^ Cambridge Nucleomics LiveLabs Babraham Research Campus Cambridge UK; ^6^ A*STAR Infectious Diseases Labs (A*STAR IDL) Agency for Science, Technology and Research (A*STAR) Singapore Singapore

**Keywords:** DNA nanotechnology, multiplexed molecular detection, programmable nanoarrays, single‐molecule sensing, solid‐state nanopores

## Abstract

Sensitive and multiplexed RNA analysis at the single‐molecule level remains a key challenge in molecular diagnostics. Conventional fluorescence microarrays provide high throughput but lack molecular resolution, whereas single‐molecule sensors such as nanopores offer exquisite precision but limited scalability. Here, we present a DNA carrier‐based nanoarray that integrates programmable nucleic acid hybridization with solid‐state nanopore readout for direct, multiplexed RNA detection. Each modular DNA carrier is pre‐assembled with spatially defined probe sites that sequence‐specifically capture RNA targets of varying lengths and conformations, in which poly(dT) sequences both structurally facilitate target binding and enhance signal strength without the need for fluorescent or protein labeling. Upon nanopore translocation, hybridized carrier‐target complexes generate binary ionic current signatures, allowing single‐molecule identification of target occupancy at each sensing site. Using a ternary coordinate encoding system, we constructed a nanoarray comprising 27 carriers with 81 addressable sensing sites, enabling simultaneous detection of multiple bacterial and viral RNA targets within a single assay directly from total RNA extracts without target‐specific isolation, amplification, or enrichment. By combining the multiplexing capability of traditional microarrays with the single‐molecule precision of nanopore sensing, this carrier‐based nanoarray establishes a scalable, programmable, and universally adaptable framework for high‐throughput molecular diagnostics in complex biological backgrounds.

## Graphical abstract

A carrier‐based DNA nanoarray integrates programmable nucleic acid hybridization with solid‐state nanopore readout for direct RNA detection at single‐molecule resolution. A ternary encoding system enables 27 carriers with 81 addressable sensing sites, allowing the simultaneous identification of targets spanning a wide size range, from short microRNAs and transfer RNAs to bacterial ribosomal RNAs and viral RNAs within a single assay.

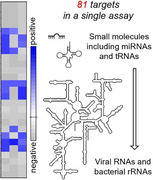

## 1 Introduction

DNA arrays [1, 2] have long served as a cornerstone of molecular sensing, enabling the parallel interrogation of nucleic acid sequences through spatially encoded hybridization patterns. Conventional microarrays rely on surface‐immobilized oligonucleotide (oligo) probes fabricated by photolithography, spotting, or inkjet printing [3, 4, 5]. While these systems have profoundly advanced gene expression profiling, their performance is hindered by surface heterogeneity and restricted probe accessibility [2, 6]. Such platforms also typically require intricate fabrication workflows and meticulous calibration to achieve uniform probe immobilization across the array, yet suffer from batch‐to‐batch variation and limited flexibility for probe customization [7, 8]. Beyond these technical constraints, microarrays have seen limited translation into clinical practice due to issues of RNA degradation, sample variability, cross‐hybridization, and the lack of standardized validation, all of which compromise analytical consistency and diagnostic reliability [8].

Recent progress in DNA nanotechnology, most notably DNA origami, has brought the concept of microarrays into the nanoscale regime, giving rise to nanoarrays [9, 10, 11, 12, 13], which are capable of organizing recognition elements with nanometer precision. By decoupling molecular recognition from fixed solid substrates, DNA origami‐based nanoarrays function in solution and thereby overcome key drawbacks of surface‐bound systems, such as uneven probe distribution, steric crowding, and diffusion barriers. They provide precise spatial control over probe arrangement and stoichiometry, enabling reproducible array construction without the need for specialized lithographic masks or printing instruments. These solution‐phase architectures have been applied to single‐molecule sensing, molecular logic operations, and cell‐surface interaction studies, showcasing the power of nanoscale spatial encoding for biomolecular analysis involving ligands, aptamers, and nucleic acids. Nevertheless, current nanoarray implementations remain constrained by intrinsic design rigidity, which impedes dynamic reconfiguration and multiplexing capabilities needed for high‐throughput molecular sensing.

Various limitations in nanoarrays have motivated the development of alternative, more modular, and programmable array architectures. In particular, carrier‐based platforms [14, 15, 16], where long DNA or RNA scaffolds template the self‐assembly of short oligos into linear duplex carriers bearing customizable sensing units, offer a promising route forward. Such systems can be fabricated under mild conditions and purified as discrete molecular entities, allowing highly reproducible array production without complex processing steps. The modular nature enables facile probe substitution to accommodate different targets, greatly enhancing the overall functional versatility. Additionally, these carriers can incorporate multiple sensing domains within the same carrier, supporting parallel detection and built‐in signal calibration through the spatial arrangement of reference elements at the single‐molecule level.

Beyond challenges in array fabrication, the difficulty in achieving precise detection of molecular interactions continues to compromise the performance of high‐throughput sensing systems. Conventional fluorescence‐based readouts [10, 11, 17], while long established as the standard for hybridization assays, are fundamentally constrained by photobleaching and background fluorescence. Moreover, the need for fluorescent labeling and enzyme‐mediated amplification can perturb native molecular conformations and complicate quantitative interpretation. Alternative techniques, such as mass spectrometry [18] (MS), surface plasmon resonance [19] (SPR), biolayer interferometry [20] (BLI), isothermal titration calorimetry [21] (ITC), and atomic force microscopy [9, 10] (AFM), offer quantitative insights into binding kinetics and affinities without labeling. However, MS and ITC are low‐throughput and ensemble‐averaged; SPR and BLI require surface tethering and lack single‐molecule resolution; AFM offers direct visualization of binding topography at nanometer precision but is inherently slow, prone to tip‐induced artifacts, and unsuitable for monitoring dynamic processes in solution. As a result, molecular heterogeneity in hybridization behavior and conformational states is often obscured.

In comparison, single‐molecule nanopore sensing provides a fundamentally different detection paradigm. When an electric potential drives nucleic acids through a nanoscale pore, each molecule generates characteristic current fluctuations that encode its sequence, structure, and binding state [22, 23]. This enables label‐free, amplification‐free readout of nucleic acid interactions in real time with single‐molecule precision. Recent advances in solid‐state nanopores have demonstrated their ability to resolve structural barcodes, RNA isoforms, and molecular complexes [24, 25, 26], suggesting the potential to integrate nanopore sensing with programmable array architectures.

Here, we introduce a carrier‐based DNA nanoarray, which is compatible with single‐molecule nanopore readout. The carriers are engineered DNA scaffolds bearing modular probe regions functionalized with poly(deoxythymidine) [poly(dT)] sequences, which both facilitate RNA capture by destabilizing secondary structures and enhance hybridization signals through increased conformational flexibility. Using structural barcoding with the DNA scaffolds, we constructed a library of 27 carriers, each containing three sensing sites, as a proof‐of‐concept demonstration of multiplex RNA detection with a design capacity of 81 targets in a single assay. Nanopore translocation readout directly decodes these hybridization patterns as distinct ionic current signatures, bridging the scalability of microarrays with the molecular precision of nanopore sensing and overcoming the averaging limitations inherent to optical or label‐free ensemble measurements.

## 2 Results

### 2.1 Characterization of the Poly(dT) Sensing Element

**FIGURE 1:**
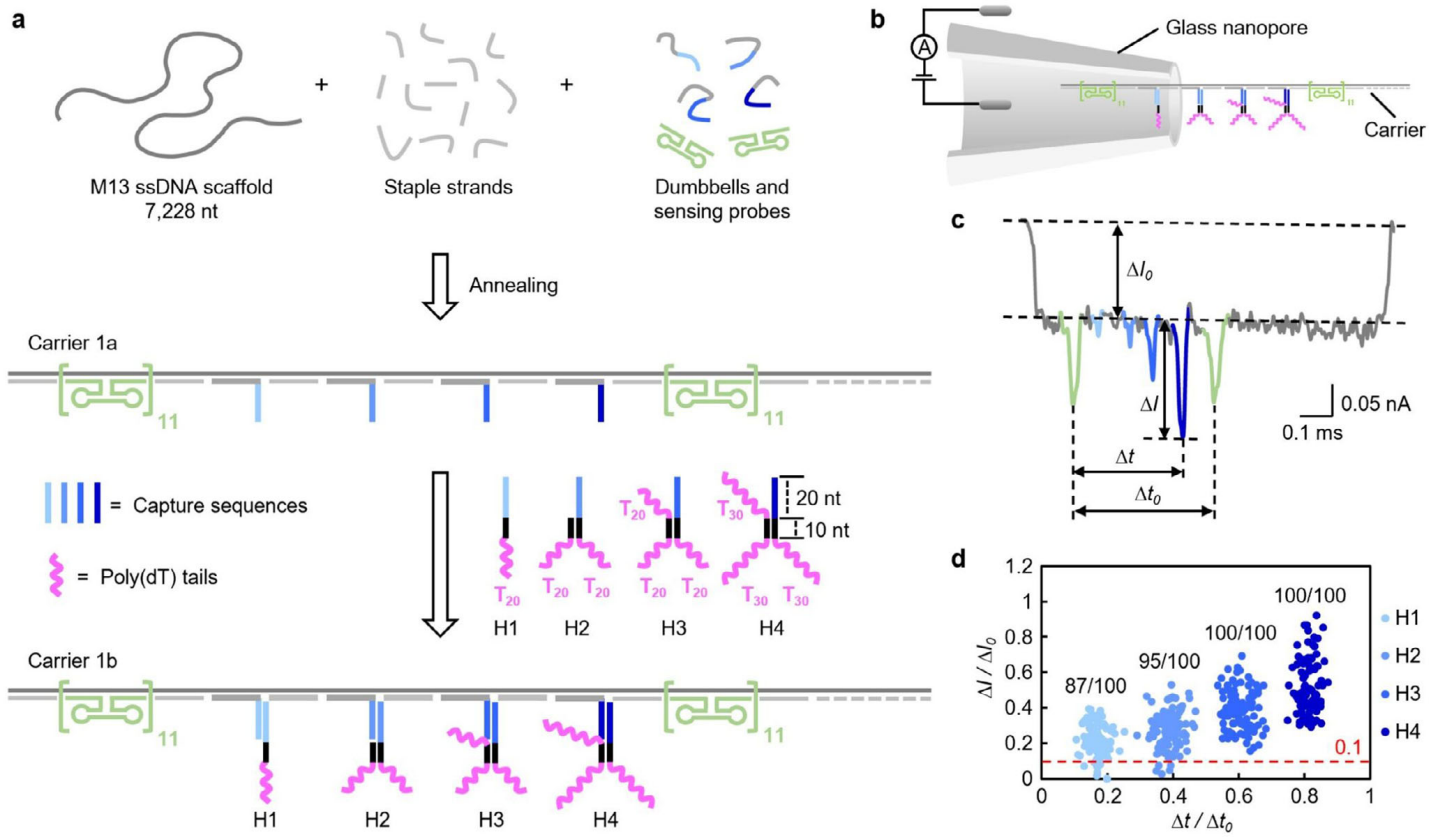
Characterization of the poly(dT) sensing element for solution‐phase carrier‐based nanoarrays in nanopore sensing. (a) Schematic illustration of the self‐assembly of an M13mp18 DNA carrier, featuring two 11‐dumbbell reference structures (green) and four sensing probes (blue), along with its interaction with poly(dT)‐enhanced target structures (H1–H4). The nanostructures H1 to H4, each containing poly(dT) tails (pink) of varying numbers and lengths, are designed to hybridize with corresponding sensing probes on the carrier. T_20_ and T_30_ denote 20 and 30 nt poly(dT) tails, respectively. Carriers 1a and 1b refer to carrier states before and after nanostructure binding. (b) Schematic representation of carrier 1b translocation through a glass nanopore. All the nanopores used in this work had an estimated diameter of 10 ± 3 nm (Methods 4.4). (c) Representative ionic current trace of carrier 1b translocation through the nanopore. The normalized parameters are defined as: spike depth (Δ*I*/Δ*I*_0_) and translocation time (Δ*t*/Δ*t*_0_), where Δ*I*_0_ represents the average first‐level current drop, Δ*I* refers to the second‐level current change beyond the first‐level plateau, Δ*t*_0_ is the time between the two reference spikes, and Δ*t* measures the interval between any target spike and the terminal reference spike. Spike colors match their corresponding sensing probes shown in (a). (d) Quantitative analysis of nanopore measurements for carrier 1b, presented as a scatterplot of Δ*I*/Δ*I*_0_ versus Δ*t*/Δ*t*_0_. The red dashed line (0.1) indicates the detection threshold required for reliable spike identification above background noise. Detection rates for each structure are shown as fractions of above‐threshold signals divided by the total 100 events analyzed.

Accurate signal generation in carrier‐based platforms critically depends on the design of the molecular sensing element. In glass nanopore readouts, the detection of small nucleic acids or molecular structures remains challenging because the analytes are typically considerably smaller than the pore diameter, producing transient or low‐amplitude current modulations that are often masked by background noise. Previous studies have demonstrated that poly(dT) strands possess beneficial properties: they exhibit minimal secondary structure formation and generate larger, more consistent nanopore current signals compared to other sequences of equal length [23, 27]. To leverage these advantageous characteristics for nanopore sensing and to identify an optimal sensing module, we designed and characterized carrier 1 (Figure 1a; Figure S1a), which integrates tunable poly(T) sequences as structural enhancers. This carrier was constructed by hybridizing short DNA oligos to the linearized single‐stranded M13mp18 genome, incorporating two reference structures (green), each comprising 11 dumbbells, and four sensing probes (blue) positioned at precisely defined locations along the scaffold.

Nanostructures H1–H4 were designed with varying numbers and lengths of poly(dT) tails (Figure 1a, pink) and hybridized to the sensing probes. Under an applied electric field, these assembled complexes translocated through the nanopore alongside carrier 1b, corresponding to carrier 1a conjugated with the target nanostructures (Figure 1b). During each translocation, the entry of the carrier backbone into the nanopore induces a primary current drop from the baseline, while the attached nanostructures generate secondary current signatures (e.g., spike amplitude and dwell time) superimposed on the first‐level drop, revealing their characteristic properties (Figure S2). The representative current trace in Figure 1c displays six discrete spikes: two corresponding to the reference structures and another four to the target structures H1–H4, with colors coded to match their respective probes on the carrier. As carriers can translocate through the nanopore in either forward or backward orientation, structures were intentionally arranged asymmetrically along the carrier to enable the determination of the translocation direction from spike distributions in the current trace.

To systematically evaluate the nanopore signal features across H1–H4, we performed quantitative analysis of 100 translocation events, presenting the result as a scatterplot of normalized spike depth (Δ*I*/Δ*I*_0_) versus normalized translocation time (Δ*t*/Δ*t*_0_) (Figure 1d). Δ*I*_0_ represents the average baseline current drop generated by the carrier backbone, Δ*I* denotes the maximum secondary current deviation within a target spike beyond the first‐level plateau, Δ*t*_0_ measures the time between two reference spikes, and Δ*t* denotes the interval between any target spike and the reference spike close to the end of the carrier (Figure 1c). A detection threshold of 0.1 (Figure 1d, red dashed line) was established as the minimum normalized current change required for reliable spike identification above background noise, which primarily arises from the inherent nanopore current fluctuations, such as thermal ionic motion, surface charge variations, and electronic noise of the measurement circuit. Data points were color‐coded by structure type, with detection rates presented as fractions of above‐threshold signals divided by the total number of translocation events analyzed (e.g., 87/100).

The results (Figure 1d) reveal a clear progression in signal quality and detection efficiency across the structures: from inconsistent spike generation with a single 20‐nt poly(dT) tail (H1), through improved but incomplete detection with dual poly(dT) tails (H2), to consistent and reliable spike generation with triple 20‐nt poly(dT) tails (H3). The optimized configuration H4, which contains three 30‐nt poly(dT) tails, achieved the highest signal‐to‐noise ratio and a 100% detection rate, with a clear separation above the Δ*I*/Δ*I*_0_ > 0.1 detection threshold.

This characterization establishes the poly(dT) module as a robust sensing element for efficient nanopore signal generation in carrier‐based nanoarrays, allowing reliable detection of small nucleic acid structures without requiring fluorescent labeling or enzymatic amplification. These poly(dT) enhancers can be functionalized with sequence‐specific capture motifs, thereby integrating target recognition and signal enhancement within a single molecular unit and making this system highly customizable and well‐suited for sequence‐sensitive applications. Additionally, the poly(dT) tail configuration, both in quantity and length, can be strategically tailored to match the size and physicochemical properties of the target molecule, enabling optimization of signal intensity while preserving structural simplicity. This design flexibility achieves a practical balance between detection sensitivity and minimalist architecture, making the platform broadly applicable across diverse nucleic acid targets.

### 2.2 Single‐Base Sensitivity Enabled by DNA Nanotechnology

**FIGURE 2:**
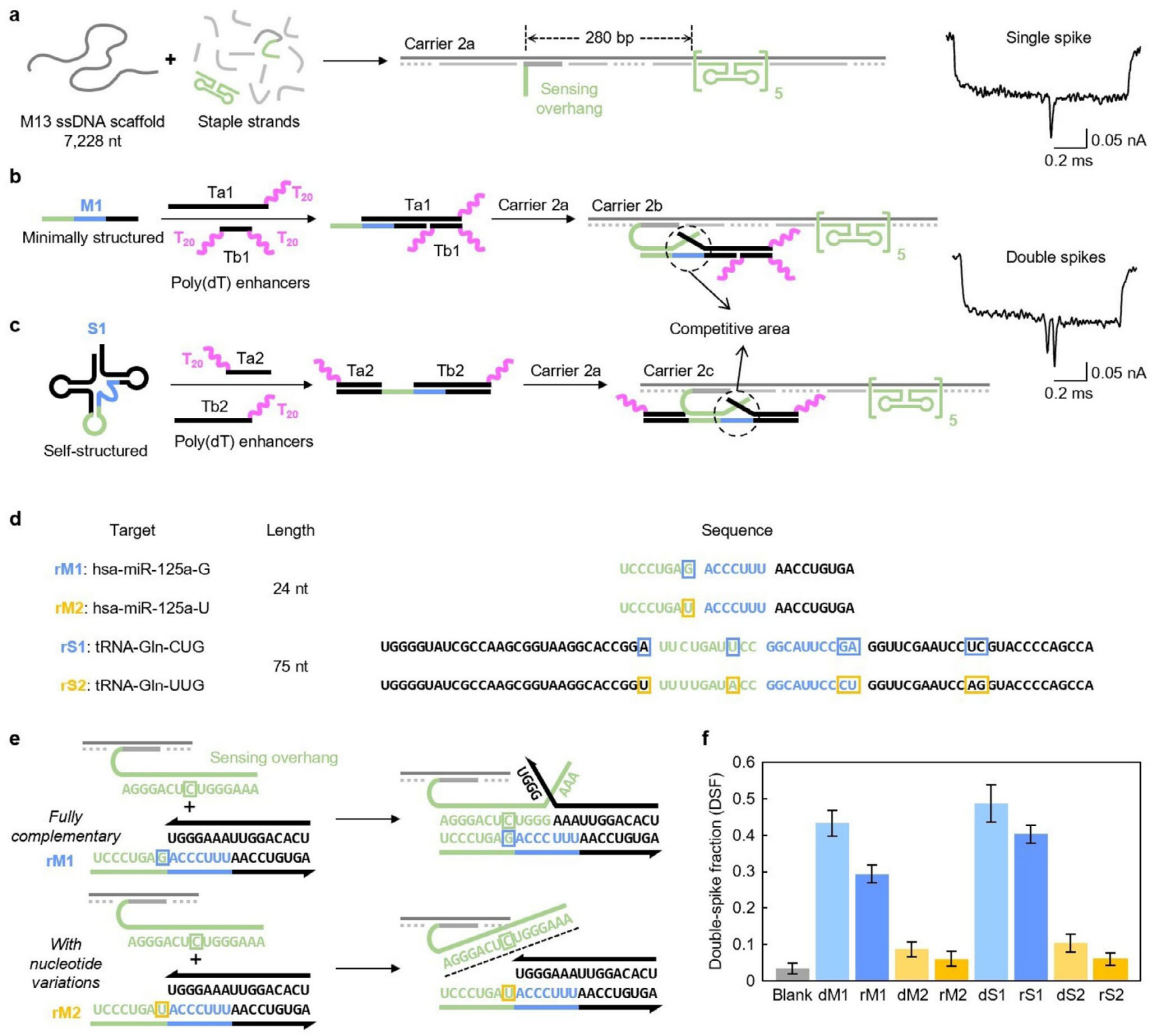
Single‐base sensitivity enabled by DNA nanotechnology on a carrier in a nanopore. (a) Schematic illustration of carrier 2a self‐assembly, featuring a sensing overhang adjacent to a five‐dumbbell reference structure. (b,c) Mechanism for detecting two types of short targets: (b) short and minimally structured targets (e.g., miRNAs) and (c) self‐structured short targets (e.g., tRNAs). RNA targets are designated as rM1 (minimally structured) and rS1 (self‐structured), with corresponding DNA analogs denoted as dM1 and dS1. The poly(dT) enhancers serve dual functions: facilitating target recognition and enhancing nanopore current signals through increased molecular size. Representative ionic current traces are shown next to each carrier configuration. The carrier designs in b and c share one representative double‐spike trace, as their translocation events appear on a comparable signal scale and show similar current patterns in our glass nanopores. (d) Sequences of miRNA and tRNA targets. rM2 and rS2 represent corresponding targets containing nucleotide variations. Sequences complementary to poly(dT) enhancers are shown in black, probe‐binding regions in green, and competitive regions in blue. DNA targets share identical sequences to their RNA counterparts but consist of DNA nucleotides. (e) Schematic illustration of the competitive binding mechanism between the carrier probe and poly(dT)‐enhanced target, using rM1 and rM2 as examples. For clarity, only the shared binding region of the enhanced target complexes is depicted. Nucleotide variations in the target sequence significantly affect probe‐target hybridization stability. (f) Quantitative analysis of the double‐spike fraction (DSF), defined as the ratio of double‐spike events to total analyzed events. The DNA carrier and target strands were used at concentrations of 0.25–5.0 nm in 15 µL, respectively, corresponding to approximately 0.6 and 1.9 ng for miRNA and tRNA targets. Error bars represent standard deviations from three independent measurements, and details are provided in Table S7.

To extend the solution‐phase carrier‐based nanoarray toward single‐base discrimination, we next investigated how DNA nanostructure engineering can enhance nanopore readout sensitivity for closely related nucleic acid targets with minimal sequence variation. Using M13mp18 ssDNA as a scaffold, we designed carrier 2a with a sensing probe positioned adjacent to a five‐dumbbell reference domain (Figure 2a; Figure S1b). Previous research has shown that five dumbbells are sufficient to produce consistent and distinguishable nanopore signals while maintaining structural simplicity [24]. During nanopore translocation, the reference structure generated a clear, single current spike, whereas the unmodified sensing probe produced barely identifiable signals (Figure 2a, right). To improve detectability for short, minimally structured targets such as microRNAs (miRNAs, 20–30 nt), we paired them with two poly(dT) enhancers, Ta1 and Tb1 (Figure 2b, left). Ta1 partly hybridized to the miRNA, while Tb1 bound to Ta1, forming a DNA complex with three 20‐nt poly(dT) tails that increased its effective translocation volume. When immobilized on carrier 2b, this structure generated a second distinct spike at the probe site adjacent to the reference spike during nanopore translocation (Figure 2b,c, right). The marked transition from a single‐ to double‐spike current signature simplifies data analysis and enhances detection reliability, as a genuine positive event is defined by the appearance of a second spike at a predetermined position relative to the reference marker. This strategy effectively minimizes false positives from nonspecific DNA knots or transient current fluctuations. Gel electrophoresis confirmed the successful assembly of poly(T)‐target complexes (Figure S3), and the optimal mixing ratio between the complex and the carrier was determined to be 20 (Figure S4). This ratio was used in all subsequent experiments throughout this study.

To accommodate larger and more structured nucleic acid targets such as transfer RNAs (tRNAs), we further refined our signal enhancement strategy to mitigate self‐folding and promote probe accessibility. Specifically, two poly(dT) enhancers (Ta2 and Tb2), each bearing a 20‐nt poly(dT) tail (Figure 2c, left), were engineered to hybridize to opposite termini of the tRNA and disrupt the internal stem‐loop regions (black), thereby destabilizing its secondary structure. This cooperative unfolding effectively exposed the target's central recognition domain (green) for efficient probe binding. A reduced number of poly(dT) tails was sufficient in this case because tRNAs are inherently larger than miRNAs, yet still incapable of producing a consistent and distinguishable nanopore signal with their native conformations. Upon successful capture of this structurally enhanced target complex by the probe on the carrier, a clear second spike again appeared adjacent to the reference signal (Figure 2b,c, right).

A key innovation in this design is the introduction of a competitive binding [28] region (Figure 2b,c, blue) within the target sequence, which establishes a dynamic equilibrium between probe binding and enhancer association. When the sensing probe and the target are perfectly complementary, hybridization is sufficiently stable to immobilize the target on the carrier upon capture, resulting in a reproducible and well‐defined nanopore signal. In contrast, for targets containing nucleotide variations, mismatches weaken the target‐probe hybridization, rendering the interaction highly susceptible to perturbations from ionic motion and thermal fluctuations in solution. These transient forces can readily displace the loosely bound target from the probe, eliminating the secondary current spike and lowering the overall double‐spike fraction (DSF, the proportion of events exhibiting the characteristic double‐spike signature among all analyzed events). This mechanism directly translates single‐base differences in sequence complementarity into measurable changes in nanopore signal patterns.

To assess base‐level specificity, we tested two variants (M1, M2) of hsa‐miR‐125a [29, 30], an miRNA associated with diseases including lung cancer and hepatitis B virus (HBV) [31, 32], and two variants (S1, S2) of *Escherichia coli* (*E. coli*) tRNA‐Gln [33, 34] as representative short nucleic acid targets (sequences in Figure 2d). DNA analogs were used for direct comparison, with deoxythymidine substituting uridine. Figure 2e illustrates the competitive binding mechanism using rM1 (miRNA target) and rM2 (miRNA variant) as examples. Analysis of the nanopore measurement results (Figure 2f) demonstrates that even a single‐nucleotide variation in the target sequence markedly reduced the DSF value by approximately four‐fold. Complementary fluorescence experiments further confirmed this single‐nucleotide discrimination capability (Figure S5).

### 2.3 Detection of Fragmented Long RNAs

**FIGURE 3:**
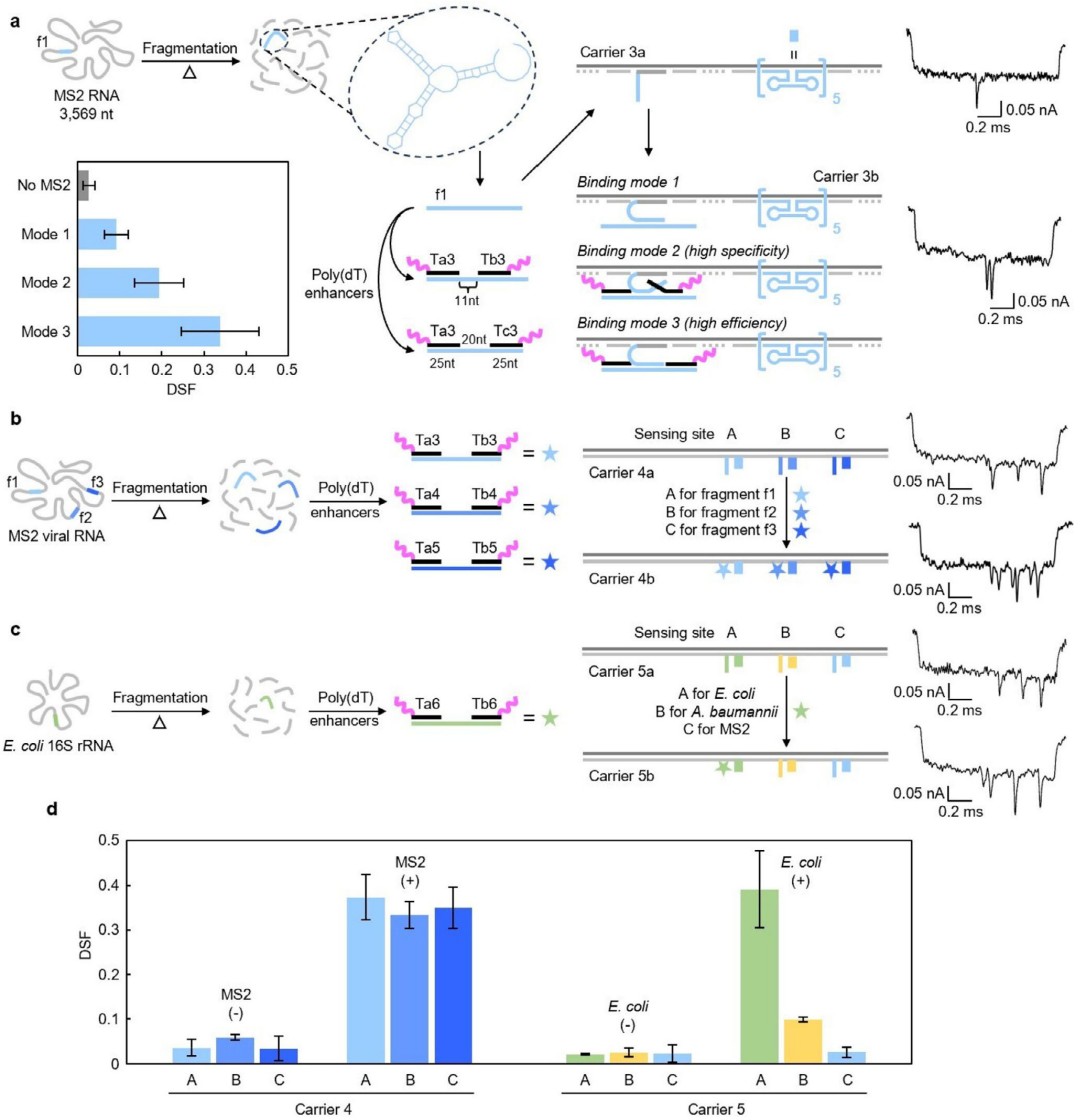
Carrier‐based detection for viral and bacterial RNA fragments using nanopore readout. An initial RNA input of 1 µg was used for each sample prior to fragmentation unless otherwise stated. (a) Schematic illustration of MS2 viral RNA fragmentation and subsequent capture of poly(dT)‐enhanced target fragments by carrier 3a. All poly(dT) tails used in this design are 20 nt in length. Three distinct binding modes between the target fragment and the sensing probe on the carrier are illustrated: mode 1 shows direct target‐probe binding without enhancement, highlighting interference from the target's secondary structure (predicted using RNAfold [37]); mode 2 depicts the competitive binding configuration where poly(dT) enhancers and the sensing probe compete for binding to the target fragment, ensuring high specificity; mode 3 demonstrates poly(dT)‐enhanced fully complementary binding, where poly(dT) enhancers facilitate target unfolding for optimal probe hybridization. DSF analysis reveals superior target capture efficiency and detection sensitivity using the fully complementary binding mode (mode 3). Further details of the nanopore measurements are provided in Table S8. (b) Simultaneous identification of three MS2 RNA fragments (f1, f2, and f3) using carrier 4, engineered with three distinct sensing sites (A, B, and C). Each fragment is targeted by specific poly(dT) enhancer pairs. (c) Selective detection of an *E. coli* 16S rRNA fragment using carrier 5 featuring three specialized sensing sites: site A for *E. coli* 16S rRNA, site B for *A. baumannii* 16S rRNA, and site C for MS2 RNA fragments. Representative ionic current traces adjacent to each carrier configuration (a–c) demonstrate characteristic signal patterns for uncaptured and captured targets. (d) Quantitative analysis of DSF across sensing sites on carriers 4 and 5, measured with and without fragmented MS2 RNA and *E. coli* 16S rRNA targets. Error bars represent standard deviations from three independent measurements, and details are provided in Table S9.

Long RNA molecules spanning kilobase lengths present distinct challenges for detection compared with short nucleic acids due to their inherent secondary structures, instability, and tendency toward self‐cleavage [35, 36]. Rather than viewing this fragmentation as a limitation, we leveraged it as an opportunity for nanoarray sensing with nanopores. To validate this concept, we first used the bacteriophage MS2 RNA as a model system. After controlled thermal fragmentation to emulate physiological degradation, we obtained RNA fragments predominantly around 100 nt in length (Figure 3a, top left, Figure S6). For conservative estimation in the sensing design, a target length of 70 nt was used. While these fragments could in principle be captured directly by carrier probes (Figure 3a, binding mode 1), random cleavage generated varied fragment lengths and sequences that might adopt stable secondary conformations (e.g., fragment f1 predicted by RNAfold [37]), reducing probe accessibility and preventing efficient hybridization. Consequently, both capture efficiency and detection rate were low.

To address this issue, we adopted a competitive enhancement strategy similar to that used for short RNA targets. Each RNA fragment was hybridized with two poly(dT) enhancers (Ta3 and Tb3) and subsequently captured by corresponding sensing probes on carrier 3a (Figure 3a, binding mode 2 “high specificity”). Successful immobilization of the poly(dT)‐enhanced RNA fragments was evidenced by the appearance of a distinct second spike in the nanopore current trace (Figure 3a, right). However, because the fragmented targets typically exhibit substantial sequence heterogeneity, precise nucleotide‐level discrimination was not required. Instead, further improvement could be achieved by optimizing sensitivity and hybridization efficiency. Accordingly, we modified the design by introducing an open binding region flanked by two poly(dT) enhancers (Ta3 and Tc3), allowing full hybridization between the target and probe (Figure 3a, binding mode 3 “high sensitivity”). This configuration proved more effective for fragment capture and yielded higher DSF values. Overall, the modular nature of this strategy enables flexible adjustment between sensitivity and specificity: a wider binding region increases capture probability and identification sensitivity, whereas a narrower binding region enhances sequence specificity. Such versatility enables tailored optimization for diverse experimental needs, supporting reliable adaptation across different detection scenarios.

Building on this strategy, we designed carrier 4 (Figure 3b; Figure S1c), which incorporates three spatially defined sensing sites (A, B, and C) to enable multiplexed detection of three distinct RNA fragments (f1, f2, and f3) derived from the same MS2 transcript, to evaluate the consistency and reliability of the detection process. In addition to the poly(T) enhancers Ta3 and Tc3 used for f1 hybridization, we introduced Ta4 and Tc4 for f2, and Ta5 and Tc5 for f3 (Figure 3b, middle). When the corresponding fragment was successfully captured, each fragment complex, represented by a color‐coded star, produced a characteristic double‐spike signature at its designated sensing site in the nanopore current trace (Figure 3b, right), confirming simultaneous multi‐target readout within a single carrier.

Beyond viral RNA, we further extended the strategy to bacterial RNA detection. Using *E. coli* 16S rRNA as a representative target, we designed enhancers Ta6 and Tc6 for a specific fragment. Carrier 5, containing three sensing sites, was functionalized with a sensing probe selective for *E. coli* 16S rRNA fragment at site A and two additional probes targeting *Acinetobacter baumannii* (*A. baumannii*) 16S rRNA and MS2 RNA at sensing sites B and C, respectively (Figure 3c; Figure S1c). Upon introduction of *E. coli* total RNA, capture of the corresponding 16S rRNA fragment produced a clear double‐spike signal at site A in nanopore readout, whereas sites B and C retained single‐spike signatures due to lack of targets (Figure 3c, right), confirming target specificity.

Analysis of the DSFs from carriers 4 and 5 further validates high target specificity (Figure 3d). Negative controls consistently yielded low DSF values (< 0.06) across three independent measurements, indicating minimal background noise. For carrier 4, the presence of MS2 RNA fragments increased DSF values more than five‐fold across all three sensing sites, with minor site‐to‐site variation. For carrier 5, exposure to fragmented *E. coli* total RNA produced the strongest DSF increase at site A, while site B showed only a slight elevation above background, and site C remained at baseline. This moderate response at site B likely reflects the inherent sequence similarity among bacterial 16S rRNAs, which can lead to limited cross‐reactivity despite careful probe design. In this work, a DSF cutoff of 0.25 was used to distinguish positives from negatives, and under the *E. coli* condition, only site A was classified as positive. These results collectively demonstrate the robustness, high specificity, and tunable sensitivity of the carrier platform with nanopore readout for detecting both viral and bacterial RNA targets, establishing a scalable and versatile strategy for broad‐spectrum nucleic acid sensing in solution.

Notably, for the main experiments shown in this study, the initial input of MS2 RNA and bacterial total RNA was 1 µg prior to fragmentation. With a typical fragmentation efficiency of 50%–60%, this input was sufficient to achieve final nanopore measurement concentrations of 0.25 nm DNA carrier and 5.0 nm target strands, enabling the collection of hundreds of linear events within 1 h. To further evaluate the sensitivity and specificity of the method under reduced‐input conditions, additional experiments were performed using the design of carrier 5, with details provided in Note S1. In these experiments, lower RNA inputs led to reduced carrier and target concentrations during nanopore measurement and therefore required longer acquisition times to obtain sufficient events for statistical analysis. Nevertheless, previous studies have shown that as few as 9 events are sufficient to yield reliable results with 99% confidence in single‐molecule nanopore measurements [26, 38]. Even at an input of 0.01 µg, with final measurement concentrations of approximately 0.008 nm for the carrier and 0.16 nm for the target, the minimum number of events required for statistical analysis could still be obtained within 2 h. As the amount of fragmented *E. coli* total RNA was reduced from 1 to 0.01 µg, the DSF value at the target sensing site showed a slight decrease but remained above the threshold of 0.25, demonstrating the robustness of the method at low input levels.

### 2.4 Nanoarray for Multiplexed Detection via a Ternary Coordinate Encoding System

Building on the array paradigm, we leveraged the carrier‐based platform to achieve multi‐site and multi‐target detection within a single molecular framework. Through the designs and nanopore measurement results of carriers 3 and 4, we demonstrated that a single carrier can effectively detect three distinct poly(dT)‐enhanced targets at sensing sites A, B, and C, analogous to spatially separated spots in a conventional microarray but integrated into a solution‐phase scaffold capable of single‐molecule characterization.

**FIGURE 4:**
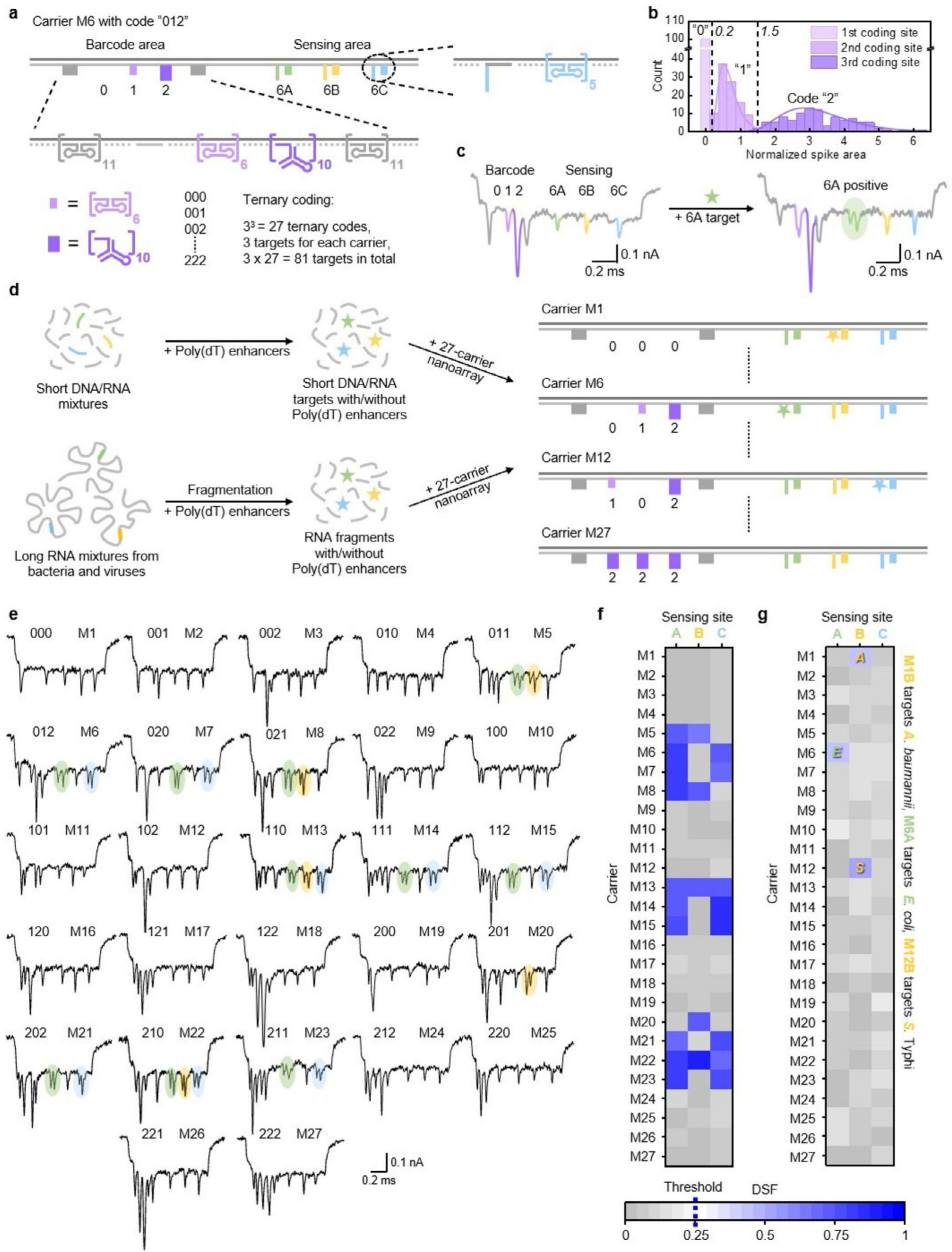
Multiplexed detection of up to 81 targets using a DNA carrier‐based nanoarray with nanopore readout. (a) Molecular design of 27 barcoded carriers (M1‐M27), each incorporating three programmable sensing sites A, B, and C. The barcode area encodes three discrete structural states: code “0” (no secondary structure), code “1” (six dumbbells), and code “2” (ten three‐way junctions). Each sensing site contains a sensing probe and a neighboring five‐dumbbell reference. (b) Distribution of normalized spike areas for current drops at the three coding sites on carrier M6. Thresholds at 0.2 and 1.5 (dashed lines) separate codes “0,” “1” and “2.” (c) Representative nanopore translocation events of carrier M6 with code “012” before (left) and after (right) incubation with 6A target RNA, corresponding to fragmented *E. coli* 16S rRNA, and its corresponding poly(dT) enhancers. (d) Schematic illustration of the simultaneous detection of multiple targets using the 27‐carrier nanoarray. In a single hybridization reaction, all targets are co‐incubated with poly(dT) enhancers and subsequently introduced to the nanoarray. Short nucleic acid targets hybridize directly with poly(dT) enhancers, while long bacterial and viral RNAs hybridize during fragmentation. Bacterial total RNAs extracted from living cells can be directly used without any further purification or amplification steps. (e) Representative nanopore translocation events for all 27 barcoded carriers following incubation with sequences chosen to create the “DNA” pattern in panel f, demonstrating barcode‐dependent identification and addressability across the carrier library. (f) Proof‐of‐concept for multiplexed detection using the carrier‐based nanoarray. The heat map shows nanopore readout of 23 DNA targets detected simultaneously across 81 sensing sites on 27 pooled carriers within a single assay. A threshold of 0.25 distinguishes positive (blue) and negative (gray) sensing sites, reconstructing the designed “DNA” pattern in blue from the nanoarray. (g) Heat map summarizing DSFs at all sensing sites across the carrier library, obtained from a single nanopore measurement with mixed poly(dT)‐enhanced fragmented long RNAs from three bacterial species, *A. baumannii*, *E. coli*, and *S*. Typhi. Each carrier was used at an initial concentration of 0.04 nm prior to nanopore measurement. Values presented are averaged from three independent measurements, and further details are provided in Tables S10 and S11.

Extending this principle, we established a ternary coordinate encoding system, in which 27 unique molecular array codes were generated by varying the number and size of secondary structural motifs positioned across three adjacent coordinates between two reference markers (Figure 4a; Figure S1d). The code “0” represents the absence of secondary structures, while codes “1” and “2” correspond to six DNA dumbbells and ten DNA three‐way junctions, respectively. This molecular encoding approach yielded a programmable sensing library comprising 27 carriers (carriers M1‐M27), each containing three sensing sites composed of a sensing probe and an adjacent five‐dumbbell reference, collectively targeting 81 nucleic acid sequences. During nanopore readout, the ternary structural code on a carrier, together with the sensing sites exhibiting characteristic double‐spike signatures on it, defines the unique identity of the carrier. Each resulting current trace could then be unambiguously mapped to a specific array coordinate, enabling accurate identification of the corresponding targets present in the sample.

To quantitatively validate the structural encoding, we analyzed the normalized distributions of spike depth and spike area (defined in Figure 1c; Figure S2c) for codes “1” and “2.” Both parameters were calibrated within the same translocation event by normalizing the spike depth and spike area of each structural code to the mean values of the two reference markers, thereby minimizing variations caused by differences in nanopore size. We found that the spike area profiles of the three structural motifs exhibited over 99% non‐overlap in nanopore translocation experiments (Figure 4b; Figure S7). Therefore, normalized spike area was adopted as a robust code identifier, with thresholds of 0.2 and 1.5 established to discriminate among the ternary states “0,” “1,” and “2.” Taking carrier M6 as an example, the nanopore translocation trace (Figure 4c, left) displayed a flat region followed by a small spike and a larger spike positioned between the two reference markers, corresponding to the ternary identity code “012.” Upon incubation with the poly(dT)‐enhanced target specific to sensing site A, nanopore readout revealed a distinct double‐spike signature only at sensing site 6A, while sensing sites 6B and 6C retained their original single‐spike signals (Figure 4c, right), confirming both code integrity and site‐specific target recognition.

Leveraging the complete carrier library, we continued to demonstrate the multiplexing capacity of this carrier‐based nanoarray system. Each carrier acted as an independent, addressable nanoarray element defined by its ternary structural code and sensing‐site composition. All targets and poly(dT) enhancers were mixed together in a single hybridization reaction and then incubated with the 27‐carrier nanoarray at room‐temperature for 1 h before nanopore measurement (Figure 4d). Short nucleic acid targets are hybridized directly with poly(dT) enhancers, whereas long bacterial or viral RNAs are annealed to poly(dT) enhancers during fragmentation, as described in previous sections.

For proof‐of‐concept, a subset of 23 targets was selected and programmed with DNA sequences assigned to predefined array coordinates (Figure S8). Representative nanopore translocation events for all 27 carriers are shown in Figure 4e. When analyzed by nanopore readout, the resulting signal pattern faithfully reconstructed the alphanumeric design “DNA,” achieved through the accurate and simultaneous detection of all 23 targets, as clearly visualized in the heat map of DSFs across 81 sensing sites distributed over 27 carriers in the library (Figure 4f; Figure S9a). A threshold of 0.25 was applied to distinguish positive from negative detections, corresponding to the presence or absence, respectively, of the target molecule at each sensing site. Sites exceeding this threshold were displayed in shades of blue, with darker tones indicating stronger responses, whereas negative sites appeared in gray. This visual representation provides an intuitive, spatially resolved summary of all detection events within the molecular nanoarray, confirming the system's ability to accurately resolve and identify multiplexed targets with high sequence specificity in a single nanopore‐based readout.

To demonstrate the applicability of this carrier‐based nanoarray for practical applications like pathogen detection, we applied the encoded sensing library to identify three representative bacterial species in a single assay: *A. baumannii*, *E. coli*, and *Salmonella enterica* serovar Typhi (S. Typhi). Specifically, carrier M1 (code “000”) targeted an *A. baumannii* 16S rRNA fragment at site B; carrier M6 (code “012”) captured an *E. coli* 16S rRNA fragment at sensing site A; and carrier M12 (code “102”) recognized an *S*. Typhi 16S rRNA fragment at site B. Following incubation of the complete 27‐carrier library with fragmented total RNA extracted from the three bacterial species, double‐spike signatures were predominantly observed at the designated sensing sites of carriers M1, M6, and M12, with DSF values exceeding the 0.25 threshold, while occasional signals at other sites remained sporadic and below the threshold, confirming the high specificity and orthogonality of detection within the multiplexed nanoarray even in complex biological backgrounds (Figure 4g; Figure S9b).

Importantly, these results were obtained using total bacterial RNA extracts without target‐specific isolation, amplification, or enrichment, demonstrating that this solution‐phase carrier‐based nanoarray maintains reliable performance even in complex biological backgrounds. This robustness highlights the system's capability for error‐tolerant, sequence‐specific molecular identification, extending the practical applicability of multiplexed RNA analysis to real‐world diagnostic contexts. By leveraging the 81 independent sensing coordinates encoded across 27 carriers, this nanoarray design is capable of supporting the simultaneous detection of up to 81 viral and bacterial RNA targets in a single assay, providing a scalable and highly parallel molecular detection platform that bridges DNA nanoarray multiplexing with single‐molecule nanopore sensing precision.

## 3 Conclusion

The carrier‐based nanoarray presented here establishes a new conceptual framework for multiplexed nucleic acid analysis, bridging the scalability of traditional microarrays with the single‐molecule precision of solid‐state nanopore sensing. Unlike surface‐immobilized arrays, this entirely solution‐phase system assembles DNA scaffolds with short oligo probes through complementary base pairing to produce discrete and stable duplex carriers that can be purified, stored, and analyzed either as individual molecular entities or as multiplexed, one‐pot detection libraries. This design ensures uniform probe distribution and controlled hybridization stoichiometry, while permitting straightforward probe replacement or reprogramming to accommodate diverse targets, allowing flexible customization without altering the fundamental carrier framework.

The nanopore readout provides direct investigation into hybridization events with single‐molecule resolution. The incorporation of poly(dT) enhancer modules improves signal‐to‐noise ratio and assists in the unfolding of structured molecules, increasing hybridization efficiency and extending the platform's compatibility to a wide range of nucleic acids. Single‐nucleotide discrimination was achieved through a competitive binding mechanism, highlighting the potential for high‐resolution variant detection. High‐specificity and high‐sensitivity binding modes were characterized using fragmented MS2 RNAs.

As a proof‐of‐concept for clinical multiplexed nucleic acid sensing applications, we demonstrated the accurate and simultaneous detection of 23 synthetic targets using a 27‐carrier nanoarray library integrating 81 sensing sites. The resulting nanopore signals faithfully reconstructed the spatially encoded pattern “DNA,” validating the programmability, robustness, and orthogonality of the nanoarray design. Moreover, the platform exhibited strong pathogen diagnostic potential, achieving selective identification of *A. baumannii*, *E. coli*, and *S. Typhi* directly from fragmented total RNA extracts without further target isolation or enrichment, underscoring its resilience to complex biological backgrounds.

From a practical perspective, the current nanopore nanoarray operates on an hour‐scale measurement window. With an input of 1 µg bacterial total RNA, a single nanopore typically produced hundreds of usable linear events within 1 h, enabling reliable analysis of multiplexed carrier mixtures, including the 27‐carrier nanoarray. For lower‐abundance targets, even at an input as low as 0.01 µg total RNA, sufficient events for statistical analysis could still be collected within 2 h. As the sample input decreases further, a longer acquisition time is required, but the detection reliability is not compromised. Future improvements in nanopore parallelization and automated signal analysis should further enhance the throughput and scalability of the platform.

While this carrier‐based nanoarray with nanopore readout represents a fundamentally different paradigm for multiplexed nucleic acid sensing, its advantages and limitations should be evaluated in the context of existing microarray and nanoarray technologies. Conventional surface‐based microarrays are a mature and standardized tool for large‐scale transcriptome profiling. However, they typically require high‐purity and relatively abundant input material in the microgram range, along with labeling or amplification steps that can introduce quantitative bias and compromise fidelity in detecting low‐abundance or structurally complex nucleic acid species. In contrast, nanopore sensing enables amplification‐free analysis at single‐molecule resolution and can detect target hybridization events with nanogram‐ or even picogram‐level target input, achieving orders‐of‐magnitude higher sensitivity. This capability is particularly advantageous for low‐yield or degraded RNA samples, such as those derived from formalin‐fixed paraffin‐embedded (FFPE) tissues, circulating extracellular vesicles, or early‐stage infection samples, where amplification is more prone to distorting transcript representation. Besides, the single‐molecule nature of nanopore sensing effectively resolves molecular heterogeneity, including conformational polymorphisms and hybridization kinetics, which remain obscured in ensemble‐averaged measurements.

Another innovation of this system is the poly(dT)‐mediated hybridization strategy, which enhances nanopore signal quality without fluorescent tagging or protein conjugation, avoiding common issues such as photobleaching, background interference, and pore clogging due to molecular aggregation [39, 40, 41, 42]. Reducing nanopore diameter offers another route to improve signal resolution, yet fabricating nucleobase‐scale pores remains technically challenging, and frequently results in unstable or ambiguous current signatures [43]. This work demonstrates poly(dT) modules as robust, nucleic acid‐only nanopore signal enhancers while remaining compatible with standard nanopore dimensions. The observed improvement in signal strength can be attributed to the fundamental physics of nanopore sensing: in a glass nanopore of fixed diameter, the magnitude of the current change directly reflects the effective volume occupied by the analyte within the sensing region. Increasing the number and length of poly(dT) tails makes the nanostructure both larger and more flexible, thereby increasing its effective volume at the nanopore tip during translocation [23].

The incorporation of poly(dT) elements also promotes efficient duplex formation across both short and long nucleic acid targets: short targets hybridize directly to poly(dT) enhancers, whereas longer bacterial or viral RNAs anneal to poly(dT) enhancer segments during fragmentation. This hybrid design enables simultaneous detection of targets with diverse lengths and structures within a single reaction, a capability that surface‐bound microarrays generally lack due to steric hindrance and diffusion constraints. In addition, this system allows flexible adjustment between an open binding configuration, which increases capture probability and detection sensitivity, and a narrower competitive binding mode that enhances sequence specificity to single‐nucleotide variation. Such tunability supports tailored optimization for a wide range of experimental needs, ensuring reliable adaptation across wide‐ranging analytical scenarios.

In terms of workflow and temporal efficiency, the carrier‐based method eliminates the need for microarray printing, surface immobilization, and optical scanning. Hybridization proceeds entirely in solution under mild conditions, while nanopore sensing provides real‐time electrical signals capable of recording thousands of translocation events per hour. As a result, the total assay time is reduced from over 20 h [44] required for conventional microarray workflows to less than 5 h from sample preparation to data acquisition. This rapid turnaround is particularly advantageous for point‐of‐care or field‐deployable diagnostic applications, where immediate minimal operation and rapid analysis are essential for timely decision‐making.

In addition to overcoming the surface‐related limitations of conventional microarrays, the carrier‐based nanoarray also offers advantages over existing DNA origami‐based nanoarray platforms [9, 10, 11, 12]. Traditional nanoarrays provide nanometer‐scale spatial precision and well‐defined probe arrangement, but their fabrication typically involves complex multistrand folding with lower yields than the linear carriers introduced in this work. Achieving high fabrication yield is essential for ensuring both the scalability and stability in solution of nanoarray platforms. Furthermore, origami scaffolds are often designed for particular structural configurations and rely on fluorescence or AFM imaging for signal readout, complicating real‐time analysis and high‐throughput applications. In comparison, the carrier‐based nanoarray employs straightforward duplex assembly to generate discrete and robust molecular entities that can be directly interrogated by nanopore sensing. This modular and reprogrammable architecture offers improved reproducibility, scalability, modular probe substitution, and compatibility with dynamic, single‐molecule readouts.

Despite these advantages, the current platform still faces several challenges. In the present study, we demonstrated a 27‐carrier nanoarray comprising 81 sensing sites, which is sufficient for proof‐of‐principle multiplexing but still far below the feature density achieved by current microarrays. Expanding the number of carriers and integrating automated electrical multiplexing will be critical for scaling the platform toward larger applications. Additionally, accurate interpretation of nanopore signals requires robust computational pipelines capable of distinguishing different hybridization‐induced signatures from one another and from background noise. Continued development of machine learning‐based signal classification and adaptive filtering algorithms will therefore be critical for translating this technology into routine analytical or diagnostic use. What's more, the current system is restricted to nucleic acid targets. Extending the carrier framework to recognize proteins, metabolites, or post‐transcriptional modifications will require new chemistries or aptamer‐based recognition elements [24, 28].

Taken together, this work establishes a new class of programmable, solution‐phase carrier‐based nanoarrays that combine the architectural control of DNA nanotechnology with the sensitivity and single‐molecule resolution of nanopore sensing. The modular framework is readily adaptable to a broad spectrum of analytes, ranging from short ones such as miRNAs and tRNAs to long sequences, including bacterial and viral transcripts. By implementing the target‐specific poly(dT) enhancement strategy, tunable for either high sensitivity or single‐nucleotide specificity, each nanoarray sensing site can be customized to maximize hybridization efficiency and signal discrimination, enabling simultaneous identification of distinct targets within a single nanopore measurement.

While not intended to replace conventional high‐density arrays for genome‐scale profiling, this platform excels in applications that demand high sensitivity, small sample input, minimal processing, and rapid, multiplexed analysis, such as targeted molecular diagnostics, clinical pathogen identification, cancer biomarker detection, and low‐copy‐number RNA assays in liquid biopsies or infectious disease screening. With continued advances in carrier design, signal analytics, and multiplexing throughput, such systems offer a scalable and universally programmable complement to existing microarray and nanoarray technologies.

## 4 Methods

### 4.1 Materials

Oligos were purchased from Integrated DNA Technologies, Inc. (IDT) and Sangon Biotech (Shanghai) Co., Ltd. M13mp18 ssDNA was purchased from Guild BioSciences. BamHI‐HF, EcoRI‐HF, Magnesium RNA Fragmentation Module, and Monarch RNA Cleanup Kit were purchased from New England Biolabs (NEB). MS2 RNA (0.8 µg/µL) was purchased from Sigma–Aldrich. *E. coli* DH5α total RNA (1 µg/µL) was purchased from Thermo Fisher Scientific. *S*. Typhi (801 ng/µL) and *A. baumannii* (592 ng/µL) total RNA was extracted from bacteria cultured in the laboratory. Other chemicals were of analytical grade and used without further purification.

### 4.2 Preparation of DNA Carriers

DNA carriers were assembled using 190 staple strands designed according to our previous work [24]. The staples were precisely mixed prior to carrier preparation. Carrier designs are detailed in Figure S1. At sensing sites, staples were replaced with dumbbells and probe strands (Tables S2 and S4). For DNA carriers containing barcodes, coding site staples were substituted with dumbbell and junction strands (Table S3).

For carrier synthesis, a 7228 nt DNA scaffold was first linearized from M13mp18 ssDNA following established protocols [24]. The reaction mixture contained 20 nm M13mp18 scaffold, 60 nm staples, and 100 nm dumbbell strands or probes. The solution was heated to 70°C and gradually cooled to 25°C over 50 min using a linear cooling ramp. The assembled carriers were purified by dilution in washing buffer (10 mm Tris‐HCl, 0.5 mm MgCl_2_, pH 8.0) to 500 µL and centrifuged using an Amicon Ultra 100 kDa filter at 9000 g for 10 min, repeated twice. The final DNA carrier solution (approximately 30 µL) was quantified using a NanoDrop 2000 spectrophotometer.

### 4.3 RNA Fragmentation and Cleanup

MS2 RNA and bacterial total RNA were fragmented using the Magnesium RNA Fragmentation Module. An initial RNA input of 1 µg was used for each sample prior to fragmentation unless otherwise stated. The fragmentation reaction was prepared in a sterile PCR tube by combining the RNA sample, poly(dT) enhancers (1.1‐fold molar excess to RNA), and RNA fragmentation buffer. The mixture was incubated in a preheated thermal cycler at 94°C for 5 min. Following incubation, the reaction was immediately terminated by transferring the tube to ice and adding RNA fragmentation stop solution. The fragmented RNA solution was then washed using the Monarch RNA Cleanup Kit following the manufacturer's protocol to remove magnesium ions and eluted in nuclease‐free water.

### 4.4 Nanopore Fabrication

Glass nanopores were fabricated using laser‐assisted pulling (P‐2000, Sutter Instrument) of quartz capillaries (outer diameter 0.5 mm and inner diameter 0.2 mm, Sutter Instrument). The formed tips, each containing a nanopore at the sharp end, were integrated into a custom PDMS chip. Before sample introduction, the chips were plasma‐cleaned for 5 min to render the surface hydrophilic, followed by immediate filling with 4 m LiCl buffer (1×TE, pH 9.0). Ag/AgCl electrodes were used to establish the electrical circuit across the nanopore.

Prior to nanopore measurements, individual nanopores were characterized by recording current–voltage (*I*–*V*) curves in 4 m LiCl buffer over a voltage range from ‐600 to 600 mV. Nanopores with a current of around 10 ± 3 nA and a root‐mean‐square (RMS) noise below 7 pA at 600 mV were selected for further experiments. The nanopore diameter was estimated based on a simplified model for conical nanopores [45]:

$$d=\frac{2I}{\pi \sigma U\tan\theta }$$

where *d* is the nanopore diameter, *I* is the measured ionic current at the applied voltage *U*, σ is the electrical conductivity of the electrolyte, and θ is the inner taper angle of the nanopore. In this work, the conductivity of 4 m LiCl was approximated as 15 S/m [46] and the inner taper angle was taken to be approximately 4° [45]. Under these conditions, the nanopore diameter could be approximated as *d* (nm) ≈ *I*_600 *mV*_ (nA).

### 4.5 Nanopore Measurement

Poly(dT) enhancers were added to miRNA and tRNA targets at a 1.1‐fold molar excess and hybridized at 75°C for 5 min, followed by a controlled cooling ramp to 4°C. For MS2 RNA and bacterial total RNA samples, poly(dT) enhancers were hybridized during RNA fragmentation. Then the targets were incubated with DNA carriers at a 20‐fold molar excess in TM buffer (10 mm Tris‐HCl, 10 mm MgCl_2_, pH 8.0) at room‐temperature for an hour. The resulting mixture was subsequently diluted in a 4 m LiCl buffer to achieve a final carrier concentration of 0.25 nm and introduced to the tip side of the glass nanopore. For multiplex detection with the 27‐carrier microarray, each carrier was used at an initial concentration of 0.04 nm prior to nanopore measurement. Electrodes were positioned on opposite sides of the nanopore, and a 600 mV potential was applied to facilitate translocation of negatively charged carrier molecules through the nanopore, generating characteristic transient current signatures.

### 4.6 Nanopore Data Analysis

Current signals were recorded using an Axopatch 200B patch clamp amplifier (Axon Instruments) at a 1 MHz sampling rate and filtered through an external low‐pass Bessel filter (Model 900CT, Frequency Devices) at 50 kHz. Signal digitization was performed using a data acquisition card (PCIe‐6251 or PCIe‐6351, National Instruments) with 16‐bit resolution. Current trace analysis was conducted using custom LabVIEW software and Python programs. Spike locations corresponding to specific structures along the event traces were predicted based on strategic staple replacements in the oligo pool design.

### 4.7 Native Polyacrylamide Gel Electrophoresis (PAGE)

To verify the successful hybridization of poly(T) enhancers with target strands (Figure S3), we performed native PAGE analysis. Poly(dT)‐enhanced target complexes were prepared by mixing 1 µm dS1 or dM1 with 1.1 µm poly(dT) enhancers in TM buffer, heating at 88°C for 5 min, and then slowly cooling to room‐temperature. The resulting DNA samples were mixed with 6× loading buffer and analyzed on a 15% native polyacrylamide gel. To examine the fragmentation of MS2 RNA and bacterial total RNA (Figure S6), the samples were subjected to the same fragmentation procedure described above, except that neither the poly(T) enhancers nor the washing step was included before directly loading onto the gel. Electrophoresis was performed in 0.5× TBE buffer (44.5 mm Tris, 44.5 mm boric acid, 1.0 mm EDTA, pH 8.0) at 110 V for 1.5 h. Gels were stained with GelRed (Biotium, Inc.) for 20 min and visualized using a UV transilluminator.

### 4.8 Fluorescence Measurement

DNA complexes DT and AC were prepared separately as described in Figure S5. Complex formation was achieved by heating strands D and T at 2 µm in TM buffer at 88°C for 5 min, followed by slow cooling to room‐temperature. Strands A and C were hybridized under identical conditions. The resulting DT and AC solutions were mixed in equal proportions and diluted to 0.1 µm in TM buffer. After 20 min of incubation, fluorescence emission spectra were collected from 553 to 700 nm using an excitation wavelength of 548 nm. To investigate mutant site effects in strand T, comparative analysis was performed by substituting strand T with single‐nucleotide variants TM1, TM2, and TM3. Strand sequences are provided in Table S6.

## Conflicts of Interest

Y.L., J.Z., and U.F.K. are co‐inventors of the ternary encoding method disclosed in the patent application CN119851760A, A Nucleic Acid Detection Method Based on a Ternary DNA Encoding Library, to which C.Y. and J.D. contributed. U.F.K. is also a co‐inventor, and J.Z. a contributor, to the nanobait method disclosed in the patent application US20240352522A1, Nucleic Acid Detection. U.F.K. and H.F.P.R. are co‐founders of Cambridge Nucleomics.

## Supporting information




**Supporting File**: advs75319‐sup‐0001‐SuppMat.docx.

## Data Availability

The data that support the findings of this study are available from the corresponding author upon reasonable request.
