# Supplementary material for: DNA Nanoarray for Multiplexed RNA Detection With Single‐Molecule Readout
Source: Adv Sci (Weinh). 2026 Apr 28;13(38):e75319. doi: 10.1002/advs.75319 (PMC13335565; doi:10.1002/advs.75319)
Supplement: Supplementary file 1 — Supporting File: advs75319‐sup‐0001‐SuppMat.docx. [file ADVS-13-e75319-s001.docx]

Supporting Information for

**DNA nanoarray for multiplexed RNA detection with single-molecule readout**

Yunxuan Li, Yesheng Wang, Chunmiao Yu, Siyu Hou, Elli Mylona, Plamena Naydenova, Jingyuan Zhao, Hong Yuan, Jingsong Duan, Hendrik F. P. Runge, Stephen Baker, Jinbo Zhu*, Ulrich F. Keyser*.

**This file includes:**

Supplementary Note S1

Supplementary Figures S1 to S9

Supplementary Tables S1 to S12

**Supplementary Note**

**Note S1. Sensitivity evaluation under reduced RNA input**

To further assess the sensitivity of the nanopore nanoarray under reduced-input conditions, we performed additional measurements using the design of carrier 5 and fragmented *E. coli* total RNA as the target source for sensing site A, while sensing sites B and C, designed for *A. baumannii* and MS2, respectively, served as false-positive controls. For the main experiments presented in the manuscript, the initial RNA input was 1 μg. Here, we systematically reduced the input amount to 0.1, 0.05, and 0.01 μg prior to fragmentation and evaluated the corresponding nanopore performance.

As shown in Table S1, a typical fragmentation recovery is 50-60%, and the target concentrations were estimated from the amount of fragmented *E. coli* total RNA under the assumption that 16S rRNA accounts for approximately 30% of total RNA^[1]^. During nanopore measurements, the carrier concentration was reduced proportionally with decreasing RNA input in order to maintain the optimized target-to-carrier ratio of 20. As summarized in Table S1, reducing the RNA input led to a lower event acquisition rate and therefore required longer measurement times to collect sufficient linear events for statistical analysis. Nevertheless, even at the lowest tested input of 0.01 μg, the minimum number of events^[2, 3]^ required for reliable analysis could still be obtained within 2 hours, and the DSF value at the target sensing site remained above the decision threshold of 0.25 while the DSF values at the non-target sensing sites B and C were clearly below this threshold, supporting the robustness of the method at low input levels.

**Table S1.** Sensitivity evaluation of carrier 5 using fragmented *E. coli* total RNA at different input amounts.

| **Initial input of E. coli total RNA before fragmentation (μg)** | **Total RNA/estimated 16S rRNA after fragmentation (ng)** | **Carrier concentration during nanopore measurement (nM)** | **Estimated target concentration during nanopore measurement (nM)** | **Linear events collected in the first hour** | **Linear events collected in the second hour** | **DSF** |
| --- | --- | --- | --- | --- | --- | --- |
| 0.1 | 61.5 / 18.5 | 0.08 | 1.6 | 46 | 43 | A: 0.42 |
|  |  |  |  |  |  | B: 0.10 |
|  |  |  |  |  |  | C: 0.01 |
| 0.05 | 24.7 / 7.4 | 0.04 | 0.8 | 26 | 28 | A: 0.39 |
|  |  |  |  |  |  | B: 0.07 |
|  |  |  |  |  |  | C: 0.04 |
| 0.01 | 5.5 / 1.7 | 0.008 | 0.16 | 4 | 5 | A: 0.33 |
|  |  |  |  |  |  | B: 0.11 |
|  |  |  |  |  |  | C: 0.11 |

**Supplementary Figures**


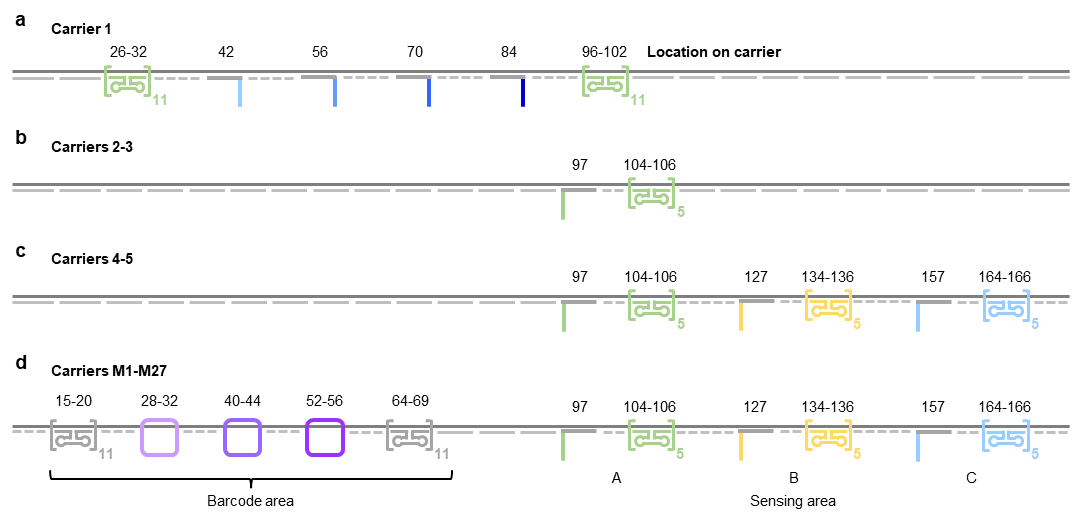


**Figure S1**. Design schematics of carriers (a) 1, (b) 2-3, (c) 4-5, and (d) M1-M27.


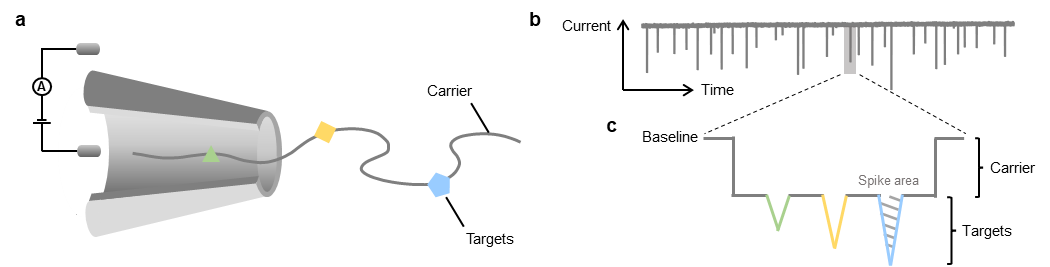


**Figure S2.** Carrier-based glass nanopore sensing. (a) Schematic illustration of a double-stranded carrier with captured target analytes translocating through a glass nanopore under an applied electrical field. (b) Representative nanopore current trace showing discrete current blockade events. Each transient decrease in current corresponds to a single carrier passing through the pore. (c) Detailed view of an individual translocation event corresponding to the carrier-target complex illustrated in (a) The entry of the carrier backbone into the nanopore causes the primary current drop from the baseline. Superimposed within this primary blockade are secondary current modulations, whose spike depth (Figure 1c) and spike area (the integrated area of the current drop) reflect the physical properties of the captured target molecules. In general, targets that displace a larger volume of solution during nanopore translocation generate more significant secondary nanopore signals, reflected in both larger spike depth and area.

**
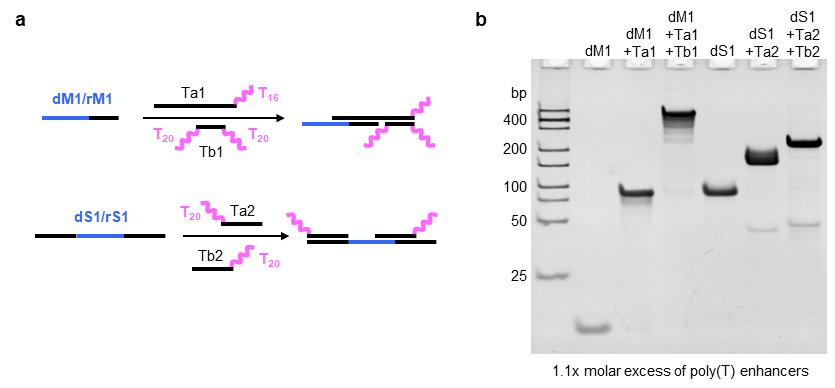
**

**Figure S3.** Verification of poly(dT)-enhanced complex formation using gel electrophoresis analysis. (a) Schematic illustration of the capture of short minimally structured (dM1/rM1) and self-structured (dS1/rS1) nucleic acid targets by poly(dT) enhancers. (b) 15% native PAGE analysis confirming the successful hybridization of poly(dT) enhancers to nucleic acid targets.


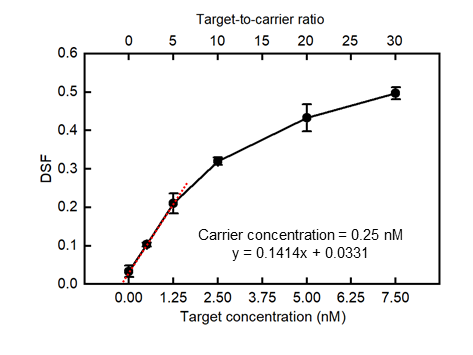


**Figure S4.** Double-spike fraction (DSF) versus poly(dT)-enhanced target concentration (taking dM1 as example) with carrier fixed at 0.25 nM, measured in nanopore. DSF increase slows beyond 20-fold target-to-carrier ratio (5 nM target concentration), hence this ratio was used throughout the study. In practical applications, the carrier concentration can be adjusted accordingly based on the target concentration to maintain this ratio. Specifically, in the low-concentration range (0-1.25 nM), the DSF shows an approximately linear dependence on target concentration. Linear fitting (red dotted line) yielded a slope of 𝑆=0.1414. Based on the standard deviation of the blank signal at 0 nM target (𝜎=0.0152), the limit of detection was estimated to be 0.35 nM using LOD=3.3𝜎/𝑆.


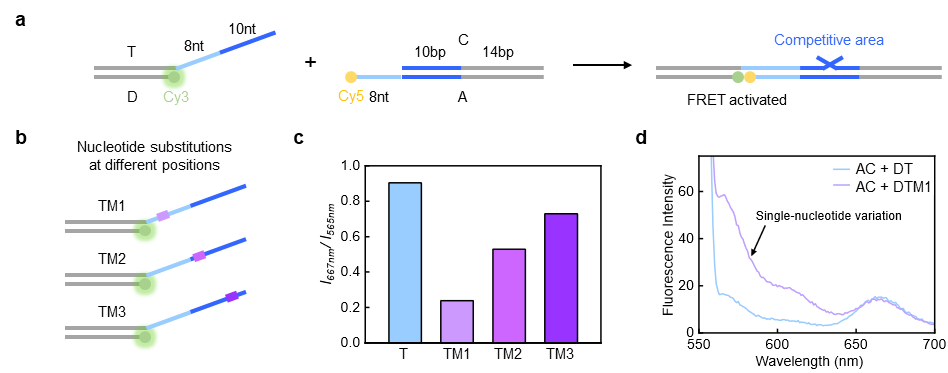


**Figure S5.** Verification of the competitive design for detection of single-nucleotide variations using fluorescence resonance energy transfer (FRET) experiments. (a) Schematic illustration of the competitive design incorporating fluorescence groups, simplified without the M13mp18 carrier context. The design consists of four key strands: T (target), D (donor), C (competitor), and A (acceptor). An 8 nt toehold region facilitates the interaction between DT and AC complexes, bringing the fluorescent donor (Cy3) and acceptor (Cy5) into close proximity. (b) Design of three variant sequences (TM1-TM3) with single-nucleotide substitutions at different positions along the T strand. Each variant introduces a strategic mismatch to test position-dependent effects on binding stability and fluorescence output. (c) Quantitative comparison of fluorescence intensity ratios (*I_667nm_ / I_565nm_*) between the perfectly matched T strand and the mutated variants TM1-TM3. The bar graph demonstrates differential responses to mismatches at various positions. TM1, containing a mismatch in the critical toehold pairing region, exhibits the most dramatic reduction in fluorescence intensity ratio (approximately 80% decrease), demonstrating the system’s highest sensitivity to mutations in this region. TM2, with a mismatch in the intermediate region, shows moderate fluorescence intensity ratio reduction (approximately 50% decrease). In contrast, TM3, harboring a mismatch in the competitive area furthest from the toehold end, displays the weakest response in fluorescence intensity ratio (approximately 30% decrease), highlighting how the impact of sequence variations diminishes with distance from the toehold region. This position-dependent sensitivity pattern aligns with the mechanistic design of the system, where toehold binding initiates the strand displacement process. (d) Fluorescence emission spectra comparing the fully complementary sequence (AC+DT) with a representative mismatch variant (AC+DTM1). The distinct spectral profiles reveal the impact of single-nucleotide variations on FRET efficiency, with the mismatched sequence showing significantly reduced energy transfer.


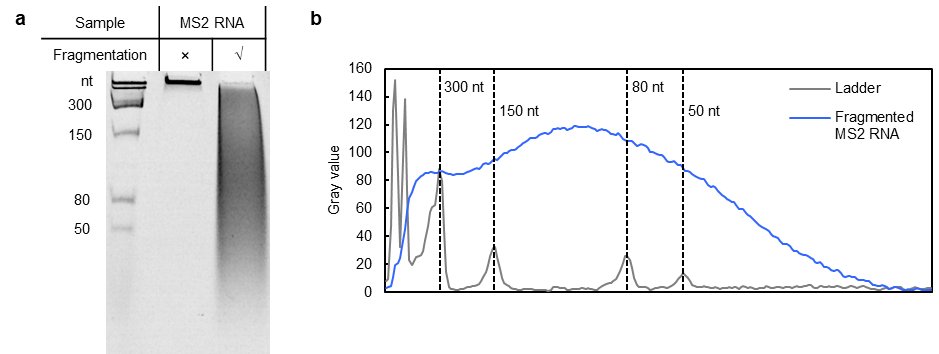


**Figure S6.** Validation of RNA fragmentation by gel electrophoresis. (a) 15% native PAGE showing the fragmentation pattern of MS2 RNA. (b) Gel band intensity analysis performed using ImageJ, indicating maximal intensity near 100 nt.


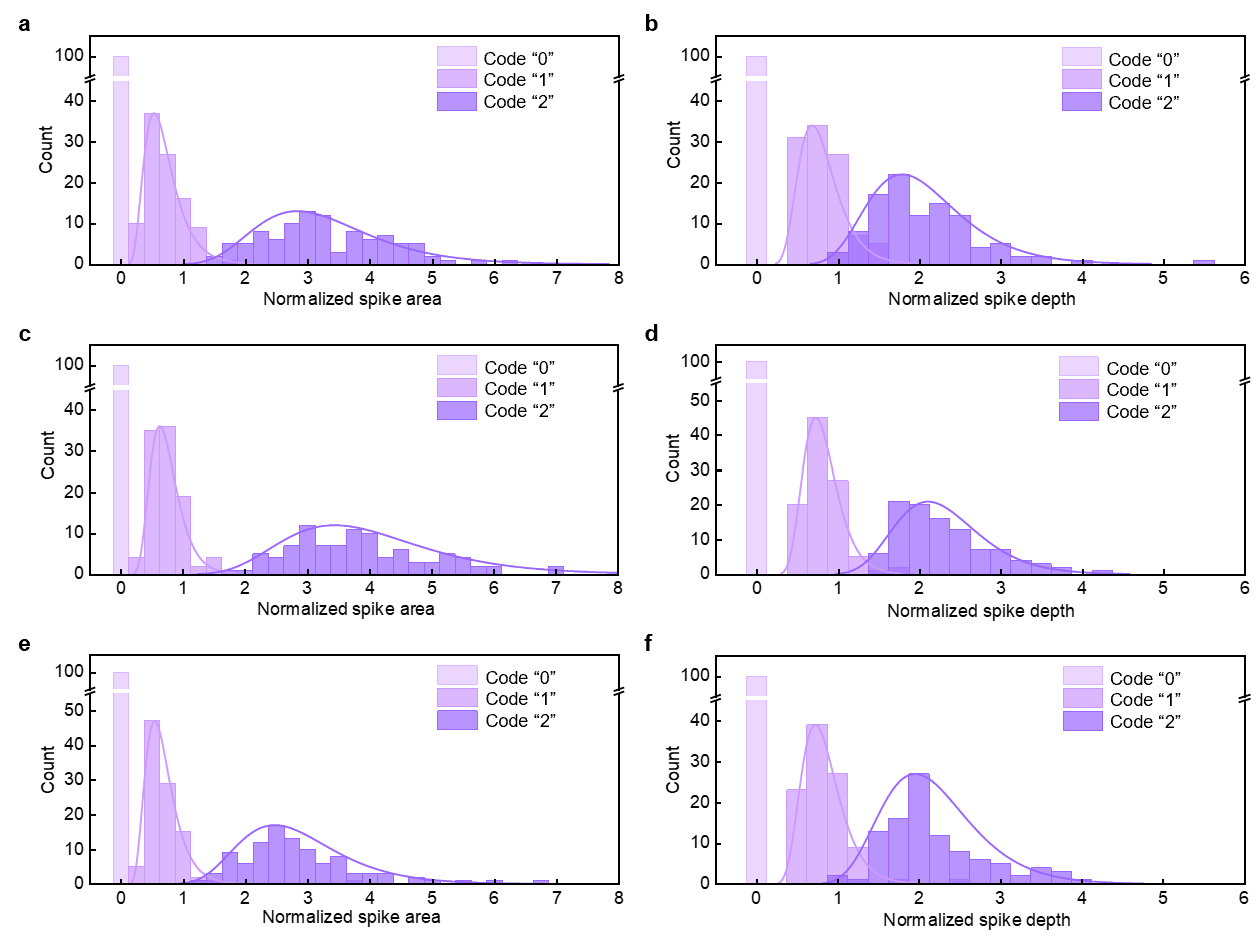


**Figure S7.** Normalized distributions of spike area and spike depth for nanopore current drops corresponding to structural codes “0”, “1”, and “2” on carrier M6. Each pair of panels (a-b, c-d, e-f) represents one replicate experiment. The spike depth distributions show overlap, making this parameter unreliable for distinguishing between codes “1” and “2”. In contrast, spike area provides clear separation among the three structural states, with threshold values of 0.2 and 1.5 effectively discriminating the ternary codes.


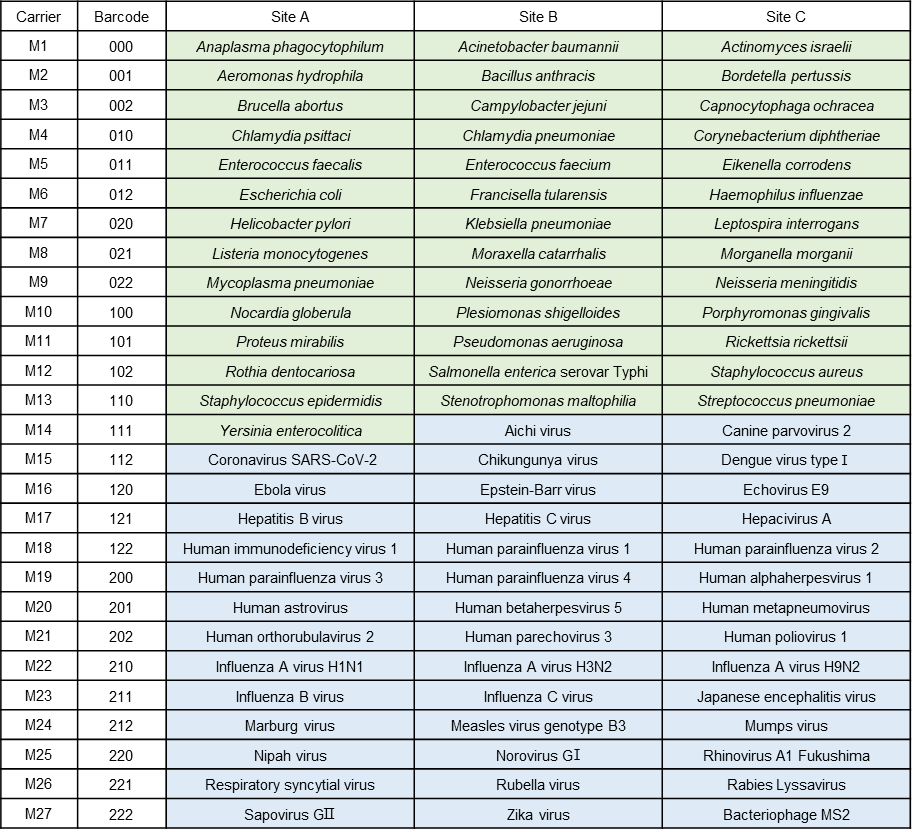


**Figure S8.** A library of 81 bacteria and viruses encoded using a ternary barcode system and three sensing sites in the carrier-based nanoarray. Bacterial species are indicated with a green background color with target sequences derived from their 16S rRNAs, while viral species are denoted by a blue background color with target sequences originating from RNA gene regions. Detailed sequences are provided in Table S5.


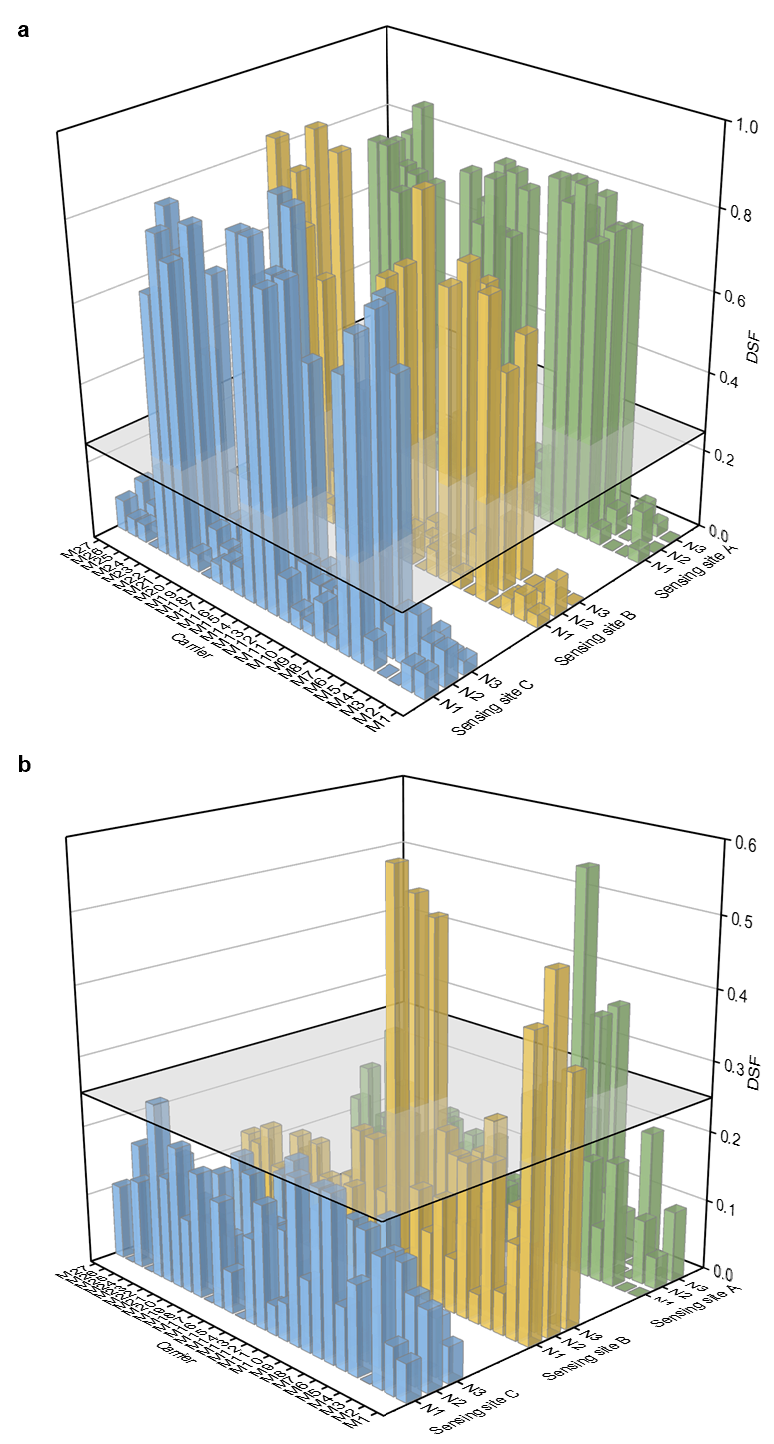


**Figure S9.** Three-dimensional bar chart representation of nanopore readouts for alphanumeric reconstruction and pathogen detection using the 27-carrier nanoarray. (a) Proof-of-concept demonstration using a subset of 23 targets programmed with DNA analog sequences corresponding to predefined array coordinates. (b) Application of the encoded carrier-based nanoarray for multiplexed bacterial detection. DSF values exceeding the threshold of 0.25 were observed at sensing site B of carrier M1, sensing site A of carrier M6, and sensing site B of carrier M12, indicating the presence of *A. baumannii*, *E. coli*, and *S.* Typhi, respectively. N1, N2, and N3 denote nanopore measurements one to three.

**Supplementary Tables**

**Table S2.** Sequences of nucleic acid targets, poly(dT) enhancers, and probes on the carriers. Sequences of the same color in each design complement each other. Varied nucleotides are highlighted in yellow.

| **Strand** | **Role** | **Sequence** |
| --- | --- | --- |
| **Verification of poly(dT) enhancement (Figure 1)** | | |
| H1 | Strand with T tail | TTTTTTTTTT TTTTTTTTTT GCTAGATGCC TCAGCCATCC  ACTAACATCC |
| PH1 | Probe on Carrier 1 for H1 | TTCGACAACT CGTATTAAAT GGATGTTAGT GGATGGCTGA  GGCATCTAGC |
| H2a | Strand with T tail | TTTTTTTTTT TTTTTTTTTT GGTAGAAGAG GTCTCATTCT  CACATCCTCA |
| H2b | Strand with T tail | CTCTTCTACC TTTTTTTTTT TTTTTTTTTT |
| PH2 | Probe on Carrier 1 for H2 | AATTAATTTT CCCTTAGAAT TGAGGATGTG AGAATGAGAC |
| H3a | Strand with T tail | TTTTTTTTTT TTTTTTTTTT TGGGTGTGAG GTTAGGAGTG  TGTTCTGGAC |
| H3b | Strand with T tail | TTTTTTTTTT TTTTTTTTTT CTCACACCCA TTTTTTTTTT  TTTTTTTTTT |
| PH3 | Probe on Carrier 1 for H3 | GAACGCGCCT GTTTATCAAC GTCCAGAACA CACTCCTAAC |
| H4a | Strand with T tail | TTTTTTTTTT TTTTTTTTTT TTTTTTTTTT GTTGGTAGTG  TGCGTTATTA TAGCTGTGCC |
| H4b | Strand with T tail | TTTTTTTTTT TTTTTTTTTT TTTTTTTTTT CACTACCAAC  TTTTTTTTTT TTTTTTTTTT TTTTTTTTTT |
| PH4 | Probe on Carrier 1 for H4 | ACACCCTGAA CAAAGTCAGA GGCACAGCTA TAATAACGCA |
| **Detection of short nucleic acid targets (Figure 2)** | | |
| rM1 | miRNA target | UCCCUGAGAC CCUUUAACCU GUGA |
| dM1 | miDNA target | TCCCTGAGAC CCTTTAACCT GTGA |
| rM2 | miRNA target | UCCCUGAUAC CCUUUAACCU GUGA |
| dM2 | miDNA target | TCCCTGATAC CCTTTAACCT GTGA |
| PS | Probe for miRNA/miDNA target | AAAGGGTCTC AGGGATTTTT GTAGCACCAT TACCATTAGC |
| Ta1 | Poly(dT) enhancer | TTTTTTTTTT TTTTTTTTTT ACCTCCTTCC TTCCTTCCTC  TCACAGGTTA AAGGGT |
| Ta1W | Enhancer without poly(dT) | ACCTCCTTCC TTCCTTCCTC TCACAGGTTA AAGGGT |
| Tb1 | Poly(dT) enhancer | TTTTTTTTTT TTTTTTTTTT GAGGAAGGAA GGAAGGAGGT  TTTTTTTTTT TTTTTTTTTT |
| Tb1W | Enhancer without poly(dT) | GAGGAAGGAA GGAAGGAGGT |
| rS1 | tRNA target | UGGGGUAUCG CCAAGCGGUA AGGCACCGGA UUCUGAUUCC  GGCAUUCCGA GGUUCGAAUC CUCGUACCCC AGCCA |
| dS1 | tDNA target | TGGGGTATCG CCAAGCGGTA AGGCACCGGA TTCTGATTCC  GGCATTCCGA GGTTCGAATC CTCGTACCCC AGCCA |
| rS2 | tRNA target | UGGGGUAUCG CCAAGCGGUA AGGCACCGGU UUUUGAUACC  GGCAUUCCCU GGUUCGAAUC CAGGUACCCC AGCCA |
| dS2 | tDNA target | TGGGGTATCG CCAAGCGGTA AGGCACCGGT TTTTGATACC  GGCATTCCCT GGTTCGAATC CAGGTACCCC AGCCA |
| PM | Probe for tRNA/tDNA target | TCGGAATGCC GGAATCAGAA TTTTT GTAGCACCAT TACCATTAGC |
| Ta2 | Poly(dT) enhancer | TCCGGTGCCT TACCGCTTGG CGATACCCCA TTTTTTTTTT  TTTTTTTTTT |
| Ta2W | Enhancer without poly(dT) | TCCGGTGCCT TACCGCTTGG CGATACCCCA |
| Tb2 | Poly(dT) enhancer | TTTTTTTTTT TTTTTTTTTT TGGCTGGGGT ACGAGGATTC  GAACCTCGGA ATGC |
| Tb2W | Enhancer without poly(dT) | TGGCTGGGGT ACGAGGATTC GAACCTCGGA ATGC |
| **Detection of fragmented long RNA targets from viruses and bacteria (Figures 3 and 4)** | | |
|  | MS2 target fragment 1 | TCGCGTTCAC AGGCTTACAA AGTAA CCTGTAGCGT TCGTCAGAGC  TCTGCGCAGA ATCGCAAATA CACCA |
| 97M1 | Probe on carrier 3a for MS2 or probe at site A on carrier 4a for MS2 | GCTCTGACGA ACGCTACAGG TTTT GTAGCACCAT TACCATTAGC |
| Ta3 | Poly(dT) enhancer | TTACTTTGTA AGCCTGTGAA CGCGA TTTTTTTTTT TTTTTTTTTT |
| Tb3 | Poly(dT) enhancer | TTTTTTTTTT TTTTTTTTTT GCGATTCTGC GCAGA GCTCTGACG |
| Tc3 | Poly(dT) enhancer | TTTTTTTTTT TTTTTTTTTT TGGTGTATTT GCGATTCTGC GCAGA |
|  | MS2 target fragment 2 | GGTTGGAGTT GCAGTTCGGT TGGTT ACCACTAATG AGTGATATCC  AGGGTGCATA TGAGATGCTT ACGAA |
| 127M2 | Probe at site B on carrier 4a for MS2 | GGATATCACT CATTAGTGGT TTTT ACCGATATAT TCGGTCGCTG |
| Ta4 | Poly(dT) enhancer | AACCAACCGA ACTGCAACTC CAACC TTTTTTTTTT TTTTTTTTTT |
| Tc4 | Poly(dT) enhancer | TTTTTTTTTT TTTTTTTTTT TTCGTAAGCA TCTCATATGC ACCCT |
|  | MS2 target fragment 3 | TTCGCTTGCG ACGATAGACT TATCG TCTGCATCCG ATTCCATCTC  CGATCGCCTG GTGTGGAGTT TTCTC |
| 157M3 | Probe at site C on carrier 4a, carrier 5a, or carrier M27 for MS2 | GAGATGGAAT CGGATGCAGA TTTT GGTCAGGATT AGAGAGTACC |
| Ta5 | Poly(dT) enhancer | CGATAAGTCT ATCGTCGCAA GCGAA TTTTTTTTTT TTTTTTTTTT |
| Tc5 | Poly(dT) enhancer | TTTTTTTTTT TTTTTTTTTT GAGAAAACTC CACACCAGGC GATCG |
|  | *E. coli* target fragment | CGACGATCCC TAGCTGGTCT GAGAG GATGACCAGC CACACTGGAA  CTGAGACACG GTCCAGACTC CTACG |
| A6 | Probe at site A on carrier 5a or carrier M6 for *E. coli* | TTCCAGTGTG GCTGGTCATC TTTT GTAGCACCAT TACCATTAGC |
| Ta6 | Poly(dT) enhancer | CTCTCAGACC AGCTAGGGAT CGTCG TTTTTTTTTT TTTTTTTTTT |
| Tc6 | Poly(dT) enhancer | TTTTTTTTTT TTTTTTTTTT CGTAGGAGTC TGGACCGTGT CTCAG |
|  | *A. Baumannii* target fragment | AATTCCAGGT GTAGCGGTGA AATGC GTAGAGATCT GGAGGAATAC  CGATGGCGAA GGCAGCCATC TGGCC |
| B1 | Probe at site B on carrier 5a or carrier M1 for *A. Baumannii* | GTATTCCTCC AGATCTCTAC TTTT ACCGATATAT  TCGGTCGCTG |
| TB1a | Poly(dT) enhancer | GCATTTCACC GCTACACCTG GAATT TTTTTTTTTT TTTTTTTTTT |
| TB1c | Poly(dT) enhancer | TTTTTTTTTT TTTTTTTTTT GGCCAGATGG CTGCCTTCGC CATCG |
|  | *S*. Typhi target fragment | GTTGTGGTTA ATAACCGCAG CAATT GACGTTACCC GCAGAAGAAG  CACCGGCTAA CTCCGTGCCA GCAGC |
| B12 | Probe at site B on carrier M12 for *S*. Typhi | CTTCTTCTGC GGGTAACGTC TTTT ACCGATATAT TCGGTCGCTG |
| TB12a | Poly(dT) enhancer | AATTGCTGCG GTTATTAACC ACAAC TTTTTTTTTT TTTTTTTTTT |
| TB12c | Poly(dT) enhancer | TTTTTTTTTT TTTTTTTTTT GCTGCTGGCA CGGAGTTAGC CGGTG |

**Table S3.** Sequences of DNA dumbbells and junctions used at barcode area. The dumbbell motifs not bound to the carrier scaffold are marked in red. The blue and yellow sequences in junction strands are complementary to matching colors in strand SJ.

| **Strand** | **Sequence** |
| --- | --- |
| **Verification of poly(dT) enhancement (Figure 1)** | |
| Dumbbell substitutes for staples 26-32 | |
| 1 | ACATCACTTG TCCTCTTTTGAGGAACAAGTTTTCTTGT CCTGAGTAGA |
| 2 | AGAACTCAAA TCCTCTTTTGAGGAACAAGTTTTCTTGT CTATCGGCCT |
| 3 | TGCTGGTAAT TCCTCTTTTGAGGAACAAGTTTTCTTGT ATCCAGAACA |
| 4 | ATATTACCGC TCCTCTTTTGAGGAACAAGTTTTCTTGT CAGCCATTGC |
| 5 | AACAGGAAAA TCCTCTTTTGAGGAACAAGTTTTCTTGT ACGCTCATGG |
| 6 | AAATACCTAC TCCTCTTTTGAGGAACAAGTTTTCTTGT ATTTTGACGC |
| 7 | TCAATCGTCT TCCTCTTTTGAGGAACAAGTTTTCTTGT GAAATGGATT |
| 8 | ATTTACATTG TCCTCTTTTGAGGAACAAGTTTTCTTGT GCAGATTCAC |
| 9 | CAGTCACACG TCCTCTTTTGAGGAACAAGTTTTCTTGT ACCAGTAATA |
| 10 | AAAGGGACAT TCCTCTTTTGAGGAACAAGTTTTCTTGT TCTGGCCAAC |
| 11 | AGAGATAGAA TCCTCTTTTGAGGAACAAGTTTTCTTGT CCCTTCTGAC |
| 12 | CTGAAAGCGT AAGAATACGT GGCACAGACA ATATTTTTGA ATGGCT |
| Dumbbell substitutes for staples 96-102 | |
| 1 | CTTGAGCCAT TCCTCTTTTGAGGAACAAGTTTTCTTGT TTGGGAATTA |
| 2 | GAGCCAGCAA TCCTCTTTTGAGGAACAAGTTTTCTTGT AATCACCAGT |
| 3 | AGCACCATTA TCCTCTTTTGAGGAACAAGTTTTCTTGT CCATTAGCAA |
| 4 | GGCCGGAAAC TCCTCTTTTGAGGAACAAGTTTTCTTGT GTCACCAATG |
| 5 | AAACCATCGA TCCTCTTTTGAGGAACAAGTTTTCTTGT TAGCAGCACC |
| 6 | GTAATCAGTA TCCTCTTTTGAGGAACAAGTTTTCTTGT GCGACAGAAT |
| 7 | CAAGTTTGCC TCCTCTTTTGAGGAACAAGTTTTCTTGT TTTAGCGTCA |
| 8 | GACTGTAGCG TCCTCTTTTGAGGAACAAGTTTTCTTGT CGTTTTCATC |
| 9 | GGCATTTTCG TCCTCTTTTGAGGAACAAGTTTTCTTGT GTCATAGCCC |
| 10 | CCTTATTAGC TCCTCTTTTGAGGAACAAGTTTTCTTGT GTTTGCCATC |
| 11 | TTTTCATAAT TCCTCTTTTGAGGAACAAGTTTTCTTGT CAAAATCACC |
| 12 | GGAACCAGAG CCACCACCGG AACCGCCTCC CTCAGAGCCG CCACCC |
| **Detection of fragmented long RNA targets from viruses and bacteria (Figures 3 and 4)** | |
| Dumbbell substitutes for staples 15-20 | |
| 1 | TTTTTGGGGT TCCTCTTTTGAGGAACAAGTTTTCTTGT CGAGGTGCCG |
| 2 | TAAAGCACTA TCCTCTTTTGAGGAACAAGTTTTCTTGT AATCGGAACC |
| 3 | CTAAAGGGAG TCCTCTTTTGAGGAACAAGTTTTCTTGT CCCCCGATTT |
| 4 | AGAGCTTGAC TCCTCTTTTGAGGAACAAGTTTTCTTGT GGGGAAAGCC |
| 5 | GGCGAACGTG TCCTCTTTTGAGGAACAAGTTTTCTTGT GCGAGAAAGG |
| 6 | AAGGGAAGAA TCCTCTTTTGAGGAACAAGTTTTCTTGT AGCGAAAGGA |
| 7 | GCGGGCGCTA TCCTCTTTTGAGGAACAAGTTTTCTTGT GGGCGCTGGC |
| 8 | AAGTGTAGCG TCCTCTTTTGAGGAACAAGTTTTCTTGT GTCACGCTGC |
| 9 | GCGTAACCAC TCCTCTTTTGAGGAACAAGTTTTCTTGT CACACCCGCC |
| 10 | GCGCTTAATG TCCTCTTTTGAGGAACAAGTTTTCTTGT CGCCGCTACA |
| 11 | GGGCGCGTAC TCCTCTTTTGAGGAACAAGTTTTCTTGT TATGGTTGCT TTGACGAG |
| Dumbbell substitutes for staples 28-31 | |
| 1 | TTGCAACAGG TCCTCTTTTGAGGAACAAGTTTTCTTGT AAAAACGCTC |
| 2 | ATGGAAATAC TCCTCTTTTGAGGAACAAGTTTTCTTGT CTACATTTTG |
| 3 | ACGCTCAATC TCCTCTTTTGAGGAACAAGTTTTCTTGT GTCTGAAATG |
| 4 | GATTATTTAC TCCTCTTTTGAGGAACAAGTTTTCTTGT ATTGGCAGAT |
| 5 | TCACCAGTCA TCCTCTTTTGAGGAACAAGTTTTCTTGT CACGACCAGT |
| 6 | AATAAAAGGG TCCTCTTTTGAGGAACAAGTTTTCTTGT ACATTCTGGC |
| 7 | CAACAGAGAT AGAACCCTTC TGACCTGAAA GC |
| Dumbbell substitutes for staples 40-43 | |
| 1 | CACTAACAAC TCCTCTTTTGAGGAACAAGTTTTCTTGT TAATAGATTA |
| 2 | GAGCCGTCAA TCCTCTTTTGAGGAACAAGTTTTCTTGT TAGATAATAC |
| 3 | ATTTGAGGAT TCCTCTTTTGAGGAACAAGTTTTCTTGT TTAGAAGTAT |
| 4 | TAGACTTTAC TCCTCTTTTGAGGAACAAGTTTTCTTGT AAACAATTCG |
| 5 | ACAACTCGTA TCCTCTTTTGAGGAACAAGTTTTCTTGT TTAAATCCTT |
| 6 | TGCCCGAACG TCCTCTTTTGAGGAACAAGTTTTCTTGT TTATTAATTT |
| 7 | TAAAAGTTTG AGTAACATTA TCATTTTGCG GA |
| Dumbbell substitutes for staples 52-55 | |
| 1 | TTACCTGAGC TCCTCTTTTGAGGAACAAGTTTTCTTGT AAAAGAAGAT |
| 2 | GATGAAACAA TCCTCTTTTGAGGAACAAGTTTTCTTGT ACATCAAGAA |
| 3 | AACAAAATTA TCCTCTTTTGAGGAACAAGTTTTCTTGT ATTACATTTA |
| 4 | ACAATTTCAT TCCTCTTTTGAGGAACAAGTTTTCTTGT TTGAATTACC |
| 5 | TTTTTTAATG TCCTCTTTTGAGGAACAAGTTTTCTTGT GAAACAGTAC |
| 6 | ATAAATCAAT TCCTCTTTTGAGGAACAAGTTTTCTTGT ATATGTGAGT |
| 7 | GAATAACCTT GCTTCTGTAA ATCGTCGCTA TT |
| Dumbbell substitutes for staples 64-69 | |
| 1 | TTACTAGAAA TCCTCTTTTGAGGAACAAGTTTTCTTGT AAGCCTGTTT |
| 2 | AGTATCATAT TCCTCTTTTGAGGAACAAGTTTTCTTGT GCGTTATACA |
| 3 | AATTCTTACC TCCTCTTTTGAGGAACAAGTTTTCTTGT AGTATAAAGC |
| 4 | CAACGCTCAA TCCTCTTTTGAGGAACAAGTTTTCTTGT CAGTAGGGCT |
| 5 | TAATTGAGAA TCCTCTTTTGAGGAACAAGTTTTCTTGT TCGCCATATT |
| 6 | TAACAACGCC TCCTCTTTTGAGGAACAAGTTTTCTTGT AACATGTAAT |
| 7 | TTAGGCAGAG TCCTCTTTTGAGGAACAAGTTTTCTTGT GCATTTTCGA |
| 8 | GCCAGTAATA TCCTCTTTTGAGGAACAAGTTTTCTTGT AGAGAATATA |
| 9 | AAGTACCGAC TCCTCTTTTGAGGAACAAGTTTTCTTGT AAAAGGTAAA |
| 10 | GTAATTCTGT TCCTCTTTTGAGGAACAAGTTTTCTTGT CCAGACGACG |
| 11 | ACAATAAACA TCCTCTTTTGAGGAACAAGTTTTCTTGT ACATGTTCAG CTAATGCA |
| Junction substitutes for staples 28-32 | |
| 1 | GGATCAGAGCTGGACG TGAGCACGAGTCTTGC TTGCAACAGGAAAAACGCT |
| 2 | GGATCAGAGCTGGACG TGAGCACGAGTCTTGC CATGGAAATACCTACATTT |
| 3 | GGATCAGAGCTGGACG TGAGCACGAGTCTTGC TGACGCTCAATCGTCTGAA |
| 4 | GGATCAGAGCTGGACG TGAGCACGAGTCTTGC ATGGATTATTTACATTGGC |
| 5 | GGATCAGAGCTGGACG TGAGCACGAGTCTTGC AGATTCACCAGTCACACGA |
| 6 | GGATCAGAGCTGGACG TGAGCACGAGTCTTGC CCAGTAATAAAAGGGACAT |
| 7 | GGATCAGAGCTGGACG TGAGCACGAGTCTTGC TCTGGCCAACAGAGATAGA |
| 8 | GGATCAGAGCTGGACG TGAGCACGAGTCTTGC ACCCTTCTGACCTGAAAGC |
| 9 | GGATCAGAGCTGGACG TGAGCACGAGTCTTGC GTAAGAATACGTGGCACAG |
| 10 | GGATCAGAGCTGGACG TGAGCACGAGTCTTGC ACAATATTTTTGAATGGCT |
| Junction substitutes for staples 40-44 | |
| 1 | GGATCAGAGCTGGACG TGAGCACGAGTCTTGC CACTAACAACTAATAGATT |
| 2 | GGATCAGAGCTGGACG TGAGCACGAGTCTTGC AGAGCCGTCAATAGATAAT |
| 3 | GGATCAGAGCTGGACG TGAGCACGAGTCTTGC ACATTTGAGGATTTAGAAG |
| 4 | GGATCAGAGCTGGACG TGAGCACGAGTCTTGC TATTAGACTTTACAAACAA |
| 5 | GGATCAGAGCTGGACG TGAGCACGAGTCTTGC TTCGACAACTCGTATTAAA |
| 6 | GGATCAGAGCTGGACG TGAGCACGAGTCTTGC TCCTTTGCCCGAACGTTAT |
| 7 | GGATCAGAGCTGGACG TGAGCACGAGTCTTGC TAATTTTAAAAGTTTGAGT |
| 8 | GGATCAGAGCTGGACG TGAGCACGAGTCTTGC AACATTATCATTTTGCGGA |
| 9 | GGATCAGAGCTGGACG TGAGCACGAGTCTTGC ACAAAGAAACCACCAGAAG |
| 10 | GGATCAGAGCTGGACG TGAGCACGAGTCTTGC GAGCGGAATTATCATCATA |
| Junction substitutes for staples 52-56 | |
| 1 | GGATCAGAGCTGGACG TGAGCACGAGTCTTGC TTACCTGAGCAAAAGAAGA |
| 2 | GGATCAGAGCTGGACG TGAGCACGAGTCTTGC TGATGAAACAAACATCAAG |
| 3 | GGATCAGAGCTGGACG TGAGCACGAGTCTTGC AAAACAAAATTAATTACAT |
| 4 | GGATCAGAGCTGGACG TGAGCACGAGTCTTGC TTAACAATTTCATTTGAAT |
| 5 | GGATCAGAGCTGGACG TGAGCACGAGTCTTGC TACCTTTTTTAATGGAAAC |
| 6 | GGATCAGAGCTGGACG TGAGCACGAGTCTTGC AGTACATAAATCAATATAT |
| 7 | GGATCAGAGCTGGACG TGAGCACGAGTCTTGC GTGAGTGAATAACCTTGCT |
| 8 | GGATCAGAGCTGGACG TGAGCACGAGTCTTGC TCTGTAAATCGTCGCTATT |
| 9 | GGATCAGAGCTGGACG TGAGCACGAGTCTTGC AATTAATTTTCCCTTAGAA |
| 10 | GGATCAGAGCTGGACG TGAGCACGAGTCTTGC TCCTTGAAAACATAGCGAT |
| Strand used for the formation of DNA junctions | |
| SJ | GCAAGACTCGTGCTCA CCGAATGCCACCACGC TTTT GCGTGGTGGCATTCGG CGTCCAGCTCTGATCC |

**Table S4.** Probe and dumbbell sequences at sensing sites. Green, yellow, and blue indicate the protruding region of probes at sensing sites A, B, and C, respectively.

| **Strand** | **Sequence** |
| --- | --- |
| Substitutes for staple 97 at sensing site A | |
| Probe A | XXXXXXXXXXXXXXXXXXXX TTTT GTAGCACCATTACCATTAGC |
| 97P | AAGGCCGGAAACGTCACC |
| Substitutes for staples 104-106 at sensing site A | |
| 1 | GCCACCAGAACCAC |
| 2 | CACCAGAGCC TCCTCTTTTGAGGAACAAGTTTTCTTGT GCCGCCAGCA |
| 3 | TTGACAGGAG TCCTCTTTTGAGGAACAAGTTTTCTTGT GTTGAGGCAG |
| 4 | GTCAGACGAT TCCTCTTTTGAGGAACAAGTTTTCTTGT TGGCCTTGAT |
| 5 | ATTCACAAAC TCCTCTTTTGAGGAACAAGTTTTCTTGT AAATAAATCC |
| 6 | TCATTAAAGC TCCTCTTTTGAGGAACAAGTTTTCTTGT CAGAATGGAA |
| Substitutes for staple 127 at sensing site B | |
| Probe B | XXXXXXXXXXXXXXXXXXXX TTTT ACCGATATATTCGGTCGCTG |
| 127P | AGGCTTGCAGGGAGTTAA |
| Substitutes for staples 134-136 at sensing site B | |
| 1 | CAAAGTACAAC TCCTCTTTTGAGGAACAAGTTTTCTTGT GGAGATTTGTAT |
| 2 | CATCGCCTGAT TCCTCTTTTGAGGAACAAGTTTTCTTGT AAATTGTGTCGA |
| 3 | AATCCGCGACC TCCTCTTTTGAGGAACAAGTTTTCTTGT TGCTCCATGTTA |
| 4 | CTTAGCCGGAA TCCTCTTTTGAGGAACAAGTTTTCTTGT CGAGGCGCAGAC |
| 5 | GGTCAATCATA TCCTCTTTTGAGGAACAAGTTTTCTTGT AGGGAACCGAA |
| Substitutes for staple 157 at sensing site C | |
| Probe C | XXXXXXXXXXXXXXXXXXXX TTTT GGTCAGGATTAGAGAGTACC |
| 157P | TTTAATTGCTCCTTTTGA |
| Substitutes for staples 164-166 at sensing site C | |
| 1 | GTGGCATCAAT TCCTCTTTTGAGGAACAAGTTTTCTTGT TCTACTAATAGT |
| 2 | AGTAGCATTAA TCCTCTTTTGAGGAACAAGTTTTCTTGT CATCCAATAAAT |
| 3 | CATACAGGCAA TCCTCTTTTGAGGAACAAGTTTTCTTGT GGCAAAGAATTA |
| 4 | GCAAAATTAAG TCCTCTTTTGAGGAACAAGTTTTCTTGT CAATAAAGCCTC |
| 5 | AGAGCATAAAG TCCTCTTTTGAGGAACAAGTTTTCTTGT CTAAATCGGTT |

**Table S5.** Target sequences for the 81 pathogens in the 27-carrier library. Bacterial sequences derived from 16S rRNA. For the proof-of-concept reconstruction of the alphanumeric design “DNA”, DNA analogues corresponding to the RNA target sequences were used. Green, yellow, and blue indicate the regions that hybridize with sensing probes at sensing sites A, B, and C, respectively. Black sequences bind to poly(T) enhancers.

| **Site** | **Target pathogen** | **Target sequence** |
| --- | --- | --- |
| 1A | *Anaplasma phagocytophilum* | GAAAAACCUU ACCACUCCUU GACAU GGAGAUUAGA UCCUUCUUAA  CGGAAGGGCG CAGUUCGGCU GGAUC |
| 1B | *Acinetobacter baumannii* | GCGAGGAGGA GGCUACUUUA GUUAA UACCUAGAGA UAGUGGACGU  UACUCGCAGA AUAAGCACCG GCUAA |
| 1C | *Actinomyces israelii* | UCAGUUCGGA UCGGUGUCUG CAACU CGACACCGUG AAGCUGGAGU  CGCUAGUAAU CGCAGAUCAG CAGUG |
| 2A | *Aeromonas hydrophila* | GGAAAGGUUG AUGCCUAAUA CGUAU CAACUGUGAC GUUACUCGCA  GAAGAAGCAC CGGCUAACUC CGUGC |
| 2B | *Bacillus anthracis* | GUGAUGAAGG CUUUCGGGUC GUAAA ACUCUGUUGU UAGGGAAGAA  CAAGUGCUAG UUGAAUAAGC UGGCA |
| 2C | *Bordetella pertussis* | GCAGGAAAGA AACGGCACGG GCUAA UAUCCUGUGC AACUGACGGU  ACCUGCAGAA UAAGCACCGG CUAAC |
| 3A | *Brucella abortus* | UGGAGACACU AUCCUUCAGU UAGGC UGGACCGGAG ACAGGUGCUG  CAUGGCUGUC GUCAGCUCGU GUCGU |
| 3B | *Campylobacter jejuni* | ACAGUUGGAA ACGACUGCUA AUACU CUAUACUCCU GCUUAACACA  AGUUGAGUAG GGAAAGUUUU UCGGU |
| 3C | *Capnocytophaga ochracea* | CUGAGCAACU GCCUUUGAAA CUGUU GGUCUUGAAU GGUUGUGAAG  UAGUUGGAAU GUGUAGUGUA GCGGU |
| 4A | *Chlamydia psittaci* | GAUGCAUACU UGAUGUGGAU AGUCU CAACCCUAUC CGUGUCGUAG  CUAACGCGUU AAGUAUGCCG CCUGA |
| 4B | *Chlamydia pneumoniae* | CGAUAUCAGC UUGUUGGUGG GGUAA AAGCCCACCA AGGCGAUGAC  GUCUAGGCGG AUUGAGAGAU UGACC |
| 4C | *Corynebacterium diphtheriae* | CACACCGCCC GUCACGUCAU GAAAG UUGGUAACAC CCGAAGCCAG  UGGCCUAACC CUUGUGGGGG GGAGC |
| 5A | *Enterococcus faecalis* | CUACAAUGGG AAGUACAACG AGUCG CUAGACCGCG AGGUCAUGCA  AAUCUCUUAA AGCUUCUCUC AGUUC |
| 5B | *Enterococcus faecium* | AUACAUGCAA GUCGAACGCU UCUUU UUCCACCGGA GCUUGCUCCA  CCGGAAAAAG AGGAGUGGCG AACGG |
| 5C | *Eikenella corrodens* | UAAACGAUGU CGAUUAGCUG UUGGG CAACUUGAUU GCUUAGUAGC  GUAGCUAACG CGUGAAAUCG ACCGC |
| 6A | *Escherichia coli* | AAAGUACUUU CAGCGGGGAG GAAGG GAGUAAAGUU AAUACCUUUG  CUCAUUGACG UUACCCGCAG AAGAA |
| 6B | *Francisella tularensis* | UGUUAAGUCA GAUGUGAAAG CCCAG GGCUCAACCU UGGAACUGCA  UUUGAUACUG GCAAACUAGA GUACG |
| 6C | *Haemophilus influenzae* | AAGGCCUUCG GGUUGUAAAG UUCUU UCGGUAUUGA GGAAGGUUGA  UGUGUUAAUA GCACAUCAAA UUGAC |
| 7A | *Helicobacter pylori* | AUACCCUGGU AGUCCACGCC CUAAA CGAUGGAUGC UAGUUGUUGG  AGGGCUUAGU CUCUCCAGUA AUGCA |
| 7B | *Klebsiella pneumoniae* | CUAACACAUG CAAGUCGAGC GGUAG CACAGAGAGC UUGCUCUCGG  GUGACGAGCG GCGGACGGGU GAGUA |
| 7C | *Leptospira interrogans* | GGUGAGUAAC ACGUGGGUAA UCUUC CUCUGAGUCU GGGAUAACUU  UCCGAAAGGG AAGCUAAUAC UGGAU |
| 8A | *Listeria monocytogenes* | CGCGAAGAAC CUUACCAGGU CUUGA CAUCCUUUGA CCACUCUGGA  GACAGAGCUU UCCCUUCGGG GACAA |
| 8B | *Moraxella catarrhalis* | UACGACCUAC GGGUGAAAGG GGGCU UUUAGCUCUC GCUAUUAGAU  GAGCCUAAGU CGGAUUAGCU GGUUG |
| 8C | *Morganella morganii* | AAGGCCUUCG GGUUGUAAAG UACUU UCAGUCGGGA GGAAGGUGUC  AAGGUUAAUA ACCUUGGCAA UUGAC |
| 9A | *Mycoplasma pneumoniae* | GCCAGCAGUC GCGGUAAUAC AUAGG UCGCAAGCGU UAUCCGGAUU  UAUUGGGCGU AAAGCAAGCG CAGGC |
| 9B | *Neisseria gonorrhoeae* | GUAAAGGACU UUUGUCAGGG AAGAA AAGGCCGUUG CCAAUAUCGG  CGGCCGAUGA CGGUACCUGA AGAAU |
| 9C | *Neisseria meningitidis* | AAAGGACUUU UGUCAGGGAA GAAAA GGCUGUUGCU AAUAUCAGCG  GCUGAUGACG GUACCUGAAG AAUAA |
| 10A | *Nocardia globerula* | GCCAGCAGCC GCGGUAAUAC GUAGG GUGCAAGCGU UGUCCGGAAU  UACUGGGCGU AAAGAGUUCG UAGGC |
| 10B | *Plesiomonas shigelloides* | GUGCUACAAU GGCAUAUACA AAGGG CGGCAAGCUA GCGAUAGUGA  GCGAAUCCCA UAAAGUAUGU CGUAG |
| 10C | *Porphyromonas gingivalis* | AACCCGUUGA AAGACGGACU AAAAC CGCAUACACU UGUAUUAUUG  CAUGAUAUUA CAAGGAAAUA UUUAU |
| 11A | *Proteus mirabilis* | AAUUCGAUGC AAUGCGAAGA ACCUU ACCUACUCUU GACAUCCAGC  GAAUCCUUUA GAGAUAGAGG AGUGC |
| 11B | *Pseudomonas aeruginosa* | AUGCAAGUCG AGCUUAUGAA GGGAG CUUGCCUUGG AUUCAGCGGC  GGACGGGUGA GUAAUGCCUA GGAAU |
| 11C | *Rickettsia rickettsii* | UGGGGAAUAU UGGACAAUGG GCGAA AGCCUGAUCC AGCAAUACCG  AGUGAGUGAU GAAGGCCUUA GGGUU |
| 12A | *Rothia dentocariosa* | UUUUAGAUGG GCUCACGGCC UAUCA GCUUGUUGGU GAGGUAAUGG  CUUACCAAGG CGACGACGGG UAGCC |
| 12B | *Salmonella enterica* serovar Typhi | AAAGUACUUU CAGCGGGGAG GAAGG UGUUGUGGUU AAUAACCGCA  GCAAUUGACG UUACCCGCAG AAGAA |
| 12C | *Staphylococcus aureus* | UUAUUAGGGA AGAACAUAUG UGUAA GUAACUGUGC ACAUCUUGAC  GGUACCUAAU CAGAAAGCCA CGGCU |
| 13A | *Staphylococcus epidermidis* | GGAAGAACAA AUGUGUAAGU AACUA UGCACGUCUU GACGGUACCU  AAUCAGAAAG CCACGGCUAA CUACG |
| 13B | *Stenotrophomonas maltophilia* | UUGUAAAGCC CUUUUGUUGG GAAAG AAAUCCAGCU GGCUAAUACC  CGGUUGGGAU GACGGUACCC AAAGA |
| 13C | *Streptococcus pneumoniae* | GAGUGGAUGU UGCAUGACAU UUGCU UAAAAGGUGC ACUUGCAUCA  CUACCAGAUG GACCUGCGUU GUAUU |
| 14A | *Yersinia enterocolitica* | ACCUACUCUU GACAUCCACG GAAUU UAGCAGAGAU GCUUCAGUGC  CUUCGGGAAC UGUGAGACAG GUGCU |
| 14B | Aichi virus | UCCUGGGCUG CCAUGUACAA CACCC ACUCCAUGUG GAAUUGCGGU  UGGCGCGUUC AAGUGACCGU CAACG |
| 14C | Canine parvovirus 2 | UUGCGCCUAA UUUAACAAAU GAAUA UGAUCCUGAU GCAUCUGCUA  AUAUGUCAAG AAUUGUAACU UACUC |
| 15A | Coronavirus SARS-CoV-2 | UUGGUGCAGG UAUAUGCGCU AGUUA UCAGACUCAG ACUAAUUCUC  CUCGGCGGGC ACGUAGUGUA GCUAG |
| 15B | Chikungunya virus | CAACGUGCGU ACCCCAUGUU UGAGG UGGAACCUAG GCAGGUCACA  CCGAAUGACC AUGCUAAUGC UAGAG |
| 15C | Dengue virus type Ⅰ | GUGGGGAUGU AAAAACCCGG GAGGC UGCAAACCAU GGAAGCUGUA  CGCAUGGGGU AGCAGACUAG UGGUU |
| 16A | Ebola virus | UGCAGAGCAA GGACUGAUAC AAUAU CCAACAGCUU GGCAAUCAGU  AGGACACAUG AUGGUGAUUU UCCGU |
| 16B | Epstein-Barr virus | UUGCCACAUG UUUUCUGGAC ACAGG ACUAACCAUG CCAUCUCUGA  UUCUAGCUCU GGCACUGCUA GCGUC |
| 16C | Echovirus E9 | ACUGGCUGCU UAUGGUGACA AUUGA GCGAUUGUUA CCAUAUAGCU  AUUGGAUUGG CCAUCCGGUG ACAAA |
| 17A | Hepatitis B virus | UCUUGUUGAC AAAAAUCCUC ACAAU ACCACAGAGU CUAGACUUGU  GGUGGACUUC UCUCAAUUUU CUAAG |
| 17B | Hepatitis C virus | CCAUAGUGGU CUGCGGAACC GGUGA GUACACCGGA AUUGCCAGGA  CGACCGGGUC CUUUCUUGGA UCAAC |
| 17C | Hepacivirus A | UCUCGGCUCG CACAUAGACG UUCUU GCUGCUAUGG CGUCUGUUUG  CUCUACUCUU GGUAUUGGUG AGGCU |
| 18A | Human immunodeficiency virus 1 | GAUUAAUAAA UUGUAACACC UCAGC CAUUACACAG GCCUGUCCAA  AGGUAUCCUU UGAGCCAAUU CCAAU |
| 18B | Human parainfluenza virus 1 | AUUCCACGGU AAACACACAC AUUAA UACACCAGCA GGAAGGACAC  ACAUCUGGCU ACUGAUUGCA ACAAC |
| 18C | Human parainfluenza virus 2 | CAGCAUCUGC GGAGAAUGUG AAGGA GAUCAUUGAG CUCUUAAAGG  GACUUGAUCU UCGCCUUCAG ACUGU |
| 19A | Human parainfluenza virus 3 | AGAGGCUUUC AGACAAGAUG GAACA GUGCAAGCAG GGCUGGUAUU  GAGCGGUGAC ACAGUGGAUC AGAUU |
| 19B | Human parainfluenza virus 4 | AGCAAGGGGC AUUAUUAUCU CUGCU UUCCUUGCAG GCCACAUCAA  UGCAGAAUCA UCUUAUGAUU GCUGC |
| 19C | Human alphaherpesvirus 1 | UUCCAGCCGG CCCUUAGAUA AGGGG GCAGUUGGUG GUCGGACGGG  UAAGUAACAG AGUCUGACUA AGGGU |
| 20A | Human astrovirus | UAGGACGCAU CUUAAUACAA CUGGG CCUAAGCCUG CGGUGUCACA  GACGAUCACA GCAACACUUG GCACC |
| 20B | Human betaherpesvirus 5 | ACACGCACGC UGGUUACCAG GCUUA ACACUAGCCA UCACAGCGUA  GUCUGGCAAC GUUAUGAUAU CUACA |
| 20C | Human metapneumovirus | UCAUUGAGUA UGGCAAAGCA UUAGG CUCAUCCUCU ACAGGCAGCA  AAGCAGAAAG UUUAUUCGUU AAUAU |
| 21A | Human orthorubulavirus 2 | ACCUAAGUGA UGGAAUCAAU CGCAA AAGCUGUUCA GUCACUGCUA  UACCAGGAGG CUGUGUCUUA UAUUG |
| 21B | Human parechovirus 3 | CCACGCUUGU GGACCUUAUG CUCAC ACAGCCAUCC UCUAGUAAGU  UUGUAAGACG UCUGAUGACG UGUGG |
| 21C | Human poliovirus 1 | GAGGGUAGUC AAUGAUCACA ACCCA ACCAAGGUGA CCUCAAAGGU  UAGAGUAUAU UUGAAACCAA AGCAC |
| 22A | Influenza A virus H1N1 | ACAAUUGGGU AAUUGCAGCG UUGCC GGAUGGAUCU UAGGAAACCC  AGAAUGCGAA UUACUGAUUU CCAAG |
| 22B | Influenza A virus H3N2 | GUAAGUCUGA AUGCAUCACU CCAAA UGGAAGCAUU CCCAAUGACA  AACCGUUCCA AAAUGUAAAC AGGAU |
| 22C | Influenza A virus H9N2 | UAGAGGCUCU CAUGGAAUGG CUAAA GACAAGACCA AUCUUGUCAU  CUCUGACUAA GGGGGUGUUA GGAUU |
| 23A | Influenza B virus | GGCCAUGAAA GCUCAGCGCU ACUAU ACUGUCUCAU GGUCAUGUAC  CUGAAUCCUG GAAAUUAUUC GAUGC |
| 23B | Influenza C virus | AAUGGUUUUG UGCUCGGCAG AUGGG AGAGAUGGUG UGGAGAUAUA  AAGACCACAA UUAUGCCUGA AAUUG |
| 23C | Japanese encephalitis virus | GGGCCUUCUG GUGAUGUUUC UGGCC ACCCAGGAGG UCCUUCGCAA  GAGGUGGACG GCCAGAUUGA CGAUU |
| 24A | Marburg virus | AGAAAGUGAU AUUAUUUGAC ACAAA UCAUCAGGUU AGUAUCUGUA  AUCAGAUAAU AGAUGCAAUA AACUC |
| 24B | Measles virus genotype B3 | UUCCACAUUG GCAUCUGAAC UCGGU AUCACUGCUG AGGAUGCAAG  GCUUGUUUCA GAGAUUGCAA UGCAU |
| 24C | Mumps virus | UGGGGACCAA CCAUUCUAGU CCAUG CAGGCGGUCA CAUUCCGACA  ACUGCAAAAC CUUUCUUCAA CUCAA |
| 25A | Nipah virus | AAGGAUAUUU GUACCAGCUA CUAAU AGUCCAGAGC UCAGAUGGGA  ACUAACAUUG UUUGCACUUG AUGUG |
| 25B | Norovirus GI | CAGUAGUUGG UGCUGCCACG GCAGU UGCCACUGCU GGCCAAGUAA  AUAUGAUUGA CCCCUGGAUU AUGAG |
| 25C | Rhinovirus A1 Fukushima | CCGCAGCCAU GGCUCAUAAA CCAAU GAGUUUGUGG UCGUAAUGAG  UAAUUGCGGG AUGGGACCGA CUACU |
| 26A | Respiratory syncytial virus | GCCUAGGCAU AAUGGGAGAG UACAG AGGUACACCG AGGAAUCAAG  AUCUAUAUGA UGCAGCAAAG GCAUA |
| 26B | Rubella virus | CCGGGGACCU GGUUGAGUAC AUUAU GAAUUACACC GGCAAUCAGC  AGUCCCGGUG GGGCCUCGGG AGCCC |
| 26C | Rabies *lyssavirus* | GGAGAUAAGA UCACCCCGGA CUCUC UCGUGGAGAU AAAGCGUACU  GAUGUAGAAG GGAAUUGGGC UUUGA |
| 27A | Sapovirus GII | CAAUGUGAAC UAUGACCAGG CUCUC GCCACCUACG AAGCGUGGUU  UAUAGGUGGU ACAGGCACCG GCCAA |
| 27B | Zika virus | UAAUCAAAUA CACAUACCAG AACAA AGUGGUUAAG GUUCUCAGAC  CAGCUGAAGG AGGAAGAACA GUAAU |
| 27C | Bacteriophage MS2 | UCGCGUUCAC AGGCUUACAA AGUAA CCUGUAGCGU UCGUCAGAGC  UCUGCGCAGA AUCGCAAAUA CACCA |

**Table S6.** Sequences of strands used in the FRET experiment (Figure S5).

| **Strand** | **Sequence** |
| --- | --- |
| A | GAGAAGTAGGAGGA AGTGTAAGAG GTGTGAGT-Cy5 |
| C | CTCTTACACT TCCTCCTACTTCTC |
| D | Cy3-AGTGGAGTTGAGTG |
| T | CACTCAACTCCACT ACTCACAC CTCTTACACT |
| TM1 | CACTCAACTCCACT ACTCACAC CTCTTACACT |
| TM2 | CACTCAACTCCACT ACTCACAC ATCTTACACT |
| TM3 | CACTCAACTCCACT ACTCACAC CTCTTAAACT |

**Table S7.** Summary of nanopore translocation experiments in Figure 2f. $\Delta I_{0}$ refers to the average of the first-level current drop across all events being analyzed.

| **Sample** | **Δ*I*_0_ (nA)** | **Total number of analyzed events** | **Number of events with double spikes** |
| --- | --- | --- | --- |
| Carrier 2a (blank) | 0.167 | 100 | 3 |
|  | 0.188 | 100 | 5 |
|  | 0.194 | 100 | 2 |
| Carrier 2a + dM1 + poly(dT) enhancers | 0.215 | 100 | 40 |
|  | 0.186 | 100 | 43 |
|  | 0.179 | 100 | 47 |
| Carrier 2a + rM1 + poly(dT) enhancers | 0.178 | 100 | 32 |
|  | 0.230 | 100 | 29 |
|  | 0.170 | 100 | 27 |
| Carrier 2a + dM2 + poly(dT) enhancers | 0.178 | 100 | 8 |
|  | 0.180 | 100 | 11 |
|  | 0.203 | 100 | 7 |
| Carrier 2a + rM2 + poly(dT) enhancers | 0.197 | 100 | 6 |
|  | 0.198 | 100 | 8 |
|  | 0.156 | 100 | 4 |
| Carrier 2a + dS1 + poly(dT) enhancers | 0.253 | 100 | 50 |
|  | 0.187 | 100 | 53 |
|  | 0.129 | 100 | 43 |
| Carrier 2a + rS1 + poly(dT) enhancers | 0.187 | 100 | 40 |
|  | 0.146 | 100 | 43 |
|  | 0.213 | 100 | 38 |
| Carrier 2a + dS2 + poly(dT) enhancers | 0.185 | 100 | 10 |
|  | 0.187 | 100 | 13 |
|  | 0.169 | 100 | 8 |
| Carrier 2a + rS2 + poly(dT) enhancers | 0.148 | 100 | 8 |
|  | 0.136 | 100 | 5 |
|  | 0.136 | 100 | 5 |

**Table S8.** Summary of nanopore translocation experiments in Figure 3a.

| **Sample** | **Δ*I*_0_ (nA)** | **Total number of analyzed events** | **Number of events with double spikes** |
| --- | --- | --- | --- |
| Carrier 3a (blank) | 0.129 | 91 | 1 |
|  | 0.127 | 88 | 3 |
|  | 0.160 | 83 | 3 |
| Carrier 3a + f1 | 0.133 | 106 | 10 |
|  | 0.184 | 50 | 6 |
|  | 0.136 | 96 | 6 |
| Carrier 3a + f1  + Ta3 + Tb3 | 0.123 | 82 | 13 |
|  | 0.135 | 156 | 38 |
|  | 0.135 | 67 | 9 |
| Carrier 3a + f1  + Ta3 + Tc3 | 0.172 | 59 | 17 |
|  | 0.192 | 107 | 49 |
|  | 0.155 | 67 | 21 |

**Table S9.** Summary of nanopore translocation experiments in Figure 3b-d.

| **Sample** | **Δ*I*_0_ (nA)** | **Total number of analyzed events** | **Number of events with double spikes at site A** | **Number of events with double spikes at site B** | **Number of events with double spikes at site C** |
| --- | --- | --- | --- | --- | --- |
| Carrier 4a (blank) | 0.173 | 53 | 2 | 3 | 1 |
|  | 0.137 | 55 | 3 | 3 | 1 |
|  | 0.154 | 61 | 1 | 4 | 4 |
| Carrier 4a + MS2 RNA fragments + poly(dT) enhancers | 0.176 | 100 | 32 | 30 | 31 |
|  | 0.192 | 100 | 38 | 34 | 34 |
|  | 0.196 | 100 | 42 | 36 | 40 |
| Carrier 5a (blank) | 0.140 | 100 | 2 | 2 | 2 |
|  | 0.151 | 83 | 2 | 3 | 4 |
|  | 0.138 | 100 | 2 | 2 | 1 |
| Carrier 5a + *E. coli* 16S rRNA fragments + poly(dT) enhancers | 0.123 | 105 | 47 | 11 | 2 |
|  | 0.169 | 105 | 30 | 10 | 4 |
|  | 0.149 | 100 | 44 | 10 | 2 |

**Table S10.** Summary of nanopore readout for the alphanumeric pattern “DNA” using the 27-carrier nanoarray. Positive sensing sites are marked in red.

| **Carrier** | **Measurement 1** | | | **Measurement 2** | | | **Measurement 3** | | |
| --- | --- | --- | --- | --- | --- | --- | --- | --- | --- |
|  | **A** | **B** | **C** | **A** | **B** | **C** | **A** | **B** | **C** |
| M1 | 1/31 | 1/31 | 2/31 | 0/22 | 2/22 | 2/22 | 0/29 | 0/29 | 1/29 |
| M2 | 0/31 | 2/31 | 2/31 | 2/22 | 1/22 | 2/22 | 1/30 | 0/30 | 1/30 |
| M3 | 0/25 | 1/25 | 1/25 | 0/21 | 0/21 | 3/21 | 2/29 | 0/29 | 2/29 |
| M4 | 1/25 | 0/25 | 0/25 | 1/15 | 0/15 | 2/15 | 0/23 | 0/23 | 0/23 |
| M5 | 20/27 | 20/27 | 2/27 | 18/24 | 13/24 | 2/24 | 17/23 | 14/23 | 0/23 |
| M6 | 26/30 | 0/30 | 23/30 | 22/25 | 1/25 | 20/25 | 16/22 | 3/22 | 14/22 |
| M7 | 22/27 | 1/27 | 18/27 | 14/18 | 2/18 | 11/18 | 27/34 | 0/34 | 27/34 |
| M8 | 25/29 | 25/29 | 1/29 | 21/25 | 19/25 | 2/25 | 18/26 | 18/26 | 4/26 |
| M9 | 1/30 | 2/30 | 3/30 | 2/20 | 0/20 | 2/20 | 2/24 | 1/24 | 0/24 |
| M10 | 1/31 | 0/31 | 1/31 | 2/23 | 0/23 | 2/23 | 1/27 | 0/27 | 0/27 |
| M11 | 1/22 | 2/22 | 1/22 | 1/24 | 1/24 | 2/24 | 2/22 | 0/22 | 1/22 |
| M12 | 1/27 | 0/27 | 3/27 | 0/25 | 0/25 | 3/25 | 2/24 | 1/24 | 2/24 |
| M13 | 26/32 | 22/32 | 25/32 | 15/23 | 16/23 | 18/23 | 21/28 | 24/28 | 16/28 |
| M14 | 18/26 | 2/26 | 23/26 | 15/23 | 1/23 | 18/23 | 18/23 | 0/23 | 21/23 |
| M15 | 21/26 | 0/26 | 23/26 | 18/25 | 0/25 | 19/25 | 23/29 | 0/29 | 27/29 |
| M16 | 1/27 | 0/27 | 2/27 | 3/16 | 0/16 | 2/16 | 0/22 | 3/22 | 1/22 |
| M17 | 2/27 | 1/27 | 2/27 | 3/22 | 2/22 | 3/22 | 1/24 | 1/24 | 2/24 |
| M18 | 1/20 | 1/20 | 1/20 | 1/21 | 1/21 | 1/21 | 1/18 | 1/18 | 2/18 |
| M19 | 1/23 | 1/23 | 0/23 | 1/21 | 2/21 | 1/21 | 0/26 | 1/26 | 0/26 |
| M20 | 2/22 | 16/22 | 1/22 | 1/24 | 14/24 | 2/24 | 1/24 | 21/24 | 2/24 |
| M21 | 14/20 | 2/20 | 15/20 | 16/22 | 1/22 | 18/22 | 13/19 | 2/19 | 13/19 |
| M22 | 21/26 | 24/26 | 21/26 | 17/23 | 19/23 | 17/23 | 20/23 | 21/23 | 11/23 |
| M23 | 21/26 | 0/26 | 17/26 | 15/19 | 0/19 | 16/19 | 27/34 | 3/34 | 26/34 |
| M24 | 1/22 | 1/22 | 0/22 | 2/19 | 0/19 | 3/19 | 3/18 | 1/18 | 2/18 |
| M25 | 0/22 | 1/22 | 1/22 | 0/18 | 2/18 | 3/18 | 1/22 | 0/22 | 1/22 |
| M26 | 2/22 | 0/22 | 1/22 | 0/18 | 0/18 | 1/18 | 3/25 | 0/25 | 3/25 |
| M27 | 0/25 | 0/25 | 2/25 | 1/19 | 1/19 | 2/19 | 1/28 | 1/28 | 1/28 |

**Table S11.** Summary of nanopore detection results for three bacteria (*A. baumannii*, *E. coli*, and *S.* Typhi) using the 27-carrier nanoarray. Positive sensing sites are marked in red.

| **Carrier** | **Measurement 1** | | | **Measurement 2** | | | **Measurement 3** | | |
| --- | --- | --- | --- | --- | --- | --- | --- | --- | --- |
|  | **A** | **B** | **C** | **A** | **B** | **C** | **A** | **B** | **C** |
| M1 | 0/19 | 8/19 | 1/19 | 2/45 | 22/45 | 5/45 | 2/20 | 7/20 | 1/20 |
| M2 | 0/15 | 2/15 | 1/15 | 3/33 | 2/33 | 4/33 | 0/25 | 0/25 | 2/25 |
| M3 | 3/17 | 1/17 | 3/17 | 0/19 | 2/19 | 3/19 | 2/10 | 1/10 | 0/10 |
| M4 | 1/13 | 3/13 | 0/13 | 0/31 | 1/31 | 5/31 | 0/17 | 1/17 | 1/17 |
| M5 | 4/24 | 1/24 | 3/24 | 3/27 | 1/27 | 2/27 | 1/14 | 2/14 | 1/14 |
| M6 | 13/23 | 5/23 | 2/23 | 10/28 | 4/28 | 5/28 | 4/11 | 0/11 | 2/11 |
| M7 | 2/13 | 1/13 | 3/13 | 2/27 | 3/27 | 5/27 | 1/16 | 4/16 | 0/16 |
| M8 | 1/13 | 2/13 | 3/13 | 2/28 | 3/28 | 2/28 | 1/5 | 1/5 | 0/5 |
| M9 | 2/10 | 1/10 | 1/10 | 6/47 | 2/47 | 9/47 | 0/8 | 0/8 | 0/8 |
| M10 | 3/13 | 2/13 | 3/13 | 4/17 | 0/17 | 3/17 | 1/11 | 2/11 | 0/11 |
| M11 | 1/19 | 1/19 | 1/19 | 1/27 | 1/27 | 2/27 | 0/19 | 4/19 | 0/19 |
| M12 | 2/24 | 14/24 | 1/24 | 0/13 | 7/13 | 3/13 | 0/8 | 4/8 | 0/8 |
| M13 | 1/23 | 3/23 | 4/23 | 3/39 | 5/39 | 5/39 | 2/15 | 2/15 | 1/15 |
| M14 | 2/25 | 5/25 | 3/25 | 1/31 | 6/31 | 4/31 | 0/12 | 0/12 | 0/12 |
| M15 | 2/14 | 1/14 | 0/14 | 2/32 | 3/32 | 3/32 | 0/11 | 0/11 | 2/11 |
| M16 | 2/17 | 2/17 | 1/17 | 0/27 | 2/27 | 5/27 | 1/9 | 0/9 | 1/9 |
| M17 | 3/20 | 1/20 | 3/20 | 2/38 | 7/38 | 1/38 | 1/13 | 2/13 | 1/13 |
| M18 | 0/11 | 0/11 | 0/11 | 2/36 | 4/36 | 3/36 | 0/9 | 1/9 | 0/9 |
| M19 | 2/17 | 1/17 | 3/17 | 6/42 | 1/42 | 7/42 | 0/11 | 0/11 | 2/11 |
| M20 | 3/29 | 0/29 | 3/29 | 1/45 | 0/45 | 4/45 | 0/30 | 1/30 | 1/30 |
| M21 | 2/15 | 1/15 | 3/15 | 1/43 | 3/43 | 5/43 | 1/17 | 0/17 | 1/17 |
| M22 | 0/20 | 0/20 | 3/20 | 2/35 | 0/35 | 5/35 | 1/14 | 1/14 | 1/14 |
| M23 | 0/20 | 2/20 | 5/20 | 2/36 | 5/36 | 3/36 | 1/17 | 2/17 | 1/17 |
| M24 | 1/13 | 2/13 | 0/13 | 1/24 | 2/24 | 3/24 | 0/10 | 1/10 | 0/10 |
| M25 | 3/16 | 2/16 | 2/16 | 2/33 | 0/33 | 2/33 | 2/14 | 0/14 | 2/14 |
| M26 | 2/15 | 2/15 | 0/15 | 2/28 | 0/28 | 3/28 | 2/9 | 0/9 | 0/9 |
| M27 | 0/19 | 1/19 | 2/19 | 0/32 | 0/32 | 5/32 | 2/25 | 0/25 | 1/25 |

**Table S12.** Summary of nanopore translocation experiments in Figure S4.

| **Concentration of poly(dT)-enhanced dS1 relative to the M13 carrier** | **Δ*I*_0_ (nA)** | **Total number of analyzed events** | **Number of events with double spikes** |
| --- | --- | --- | --- |
| 0 | 0.167 | 100 | 3 |
|  | 0.188 | 100 | 5 |
|  | 0.194 | 100 | 2 |
| 2 | 0.163 | 100 | 10 |
|  | 0.161 | 100 | 10 |
|  | 0.161 | 100 | 11 |
| 5 | 0.177 | 100 | 19 |
|  | 0.177 | 100 | 20 |
|  | 0.184 | 100 | 24 |
| 10 | 0.191 | 100 | 32 |
|  | 0.214 | 100 | 31 |
|  | 0.223 | 100 | 33 |
| 20 | 0.215 | 100 | 40 |
|  | 0.186 | 100 | 43 |
|  | 0.179 | 100 | 47 |
| 30 | 0.169 | 100 | 51 |
|  | 0.230 | 100 | 48 |
|  | 0.175 | 100 | 50 |

**References**

1. M. Burck, S. Nouaille, A. J. Carpousis, et al., “rRNA Loss Induced by Rifampicin Addition in *Escherichia coli* Reflects Extraction Artifacts Rather Than in Vivo Degradation,” *Scientific Reports* 15, (2025): 30833, <https://doi.org/10.1038/s41598-025-14966-1>.

2. J. Zhu, N. Ermann, K. Chen, and U. F. Keyser, “Image Encoding Using Multi-Level DNA Barcodes with Nanopore Readout,” *Small* 17 (2021): e2100711, <https://doi.org/10.1002/smll.202100711>.

3. N. Ermann, *Nanopore-Based Readout of Encoded DNA Nanostructures* (University of Cambridge, 2020).
